# Binary Solvent Engineering Modulates the Microstructure of Stretchable Organic Field-Effect Transistors for Highly Sensitive NO_2_ Sensing

**DOI:** 10.3390/nano15120922

**Published:** 2025-06-13

**Authors:** Xiao Jiang, Jiaqi Zeng, Linxuan Zhang, Zhen Zhang, Rongjiao Zhu

**Affiliations:** Key Laboratory of Organic Integrated Circuits Ministry of Education & Tianjin Key Laboratory of Molecular Optoelectronic Sciences, Department of Chemistry, School of Science, Tianjin University, Tianjin 300072, Chinazhzhen@tju.edu.cn (Z.Z.)

**Keywords:** organic field-effect transistors, organic semiconductors, stretchable, nitrogen dioxide, nano-restricted domain effects

## Abstract

Stretchable organic field-effect transistors (OFETs), with inherent flexibility, versatile sensing mechanisms, and signal amplification properties, provide a unique device-level solution for the real-time, in situ detection of trace gaseous pollutants. However, serious challenges remain regarding the synergistic optimization of OFET gas sensor production preparation, mechano-electrical properties, and gas-sensing performance. Although the introduction of microstructures can theoretically provide OFETs with enhanced sensing performance, the high-precision process required for microstructure fabrication limits scale-up. Herein, a straightforward hybrid solvent strategy is proposed for regulating the intrinsic microstructure of the organic semiconductor layer, with the aim of constructing an ultrasensitive PDVT-10/SEBS fully stretchable OFET NO_2_ sensor. The binary solvent system induces the formation of nanoneedle-like structures in the PDVT-10/SEBS organic semiconductor, which achieves a maximum mobility of 2.71 cm^2^ V^−1^ s^−1^, a switching current ratio generally exceeding 10^6^, and a decrease in mobility of only 30% at 100% strain. Specifically, the device exhibits a response of up to 77.9 × 10^6^ % within 3 min and a sensitivity of up to 1.4 × 10^6^ %/ppm, and it demonstrates effective interference immunity, with a response of less than 100% to nine interferences. This work paves the way for next-generation wearable smart sensors.

## 1. Introduction

With the development of industry and the improvement in quality of life, the ever-growing gas pollution has become a serious problem [1]. Nitrogen dioxide (NO_2_), as a typical secondary pollutant with a V-shaped molecular structure, mainly originates from the high-temperature combustion processes of fossil fuels, including motor vehicle exhaust, industrial emissions, and residential fuel combustion [2]. It has been reported that exposure to NO_2_ is associated with the incidence and worsening of inflammation [3,4,5], chronic obstructive pulmonary disease [6], asthma [7], respiratory diseases [8], Parkinson’s disease [9], nervous system disease [10,11,12,13], and cardiovascular diseases [14]. In light of this, it is highly desirable to quantify the burden of NO_2_ exposure and its health effects in order to assess public safety and protect individual health [15].

Various analytical techniques, such as chemiluminescent [16], resistive [17], and electrochemical sensing [18], have been employed to measure NO_2_. Among them, OFET-based sensors have attracted much attention in the gas-sensing field due to their stability [19], low cost [20], low power consumption [21], and high performance [22]. However, it has been reported that the rigid substrate of OFETs may be an obstacle to efficient device response due to higher interfacial contact resistance [23,24], restricted gas diffusion paths [25,26], and complex and variable detection environments [27,28]. Therefore, flexible/stretchable OFETs with adaptive shape control are considered a potential option for addressing the root of the problem [29,30].

As an emerging gas-sensing technology, fully stretchable OFETs are limited by the difficulty of balancing electrical, mechanical, and sensing properties; however, the introduction of microstructures provides a new solution to this problem [31,32,33,34]. Microstructure design, which provides functional materials with an enlarged specific surface area, efficient conduction paths, and higher material utilization, is considered a potential option for optimizing film morphology and enhancing the gas-sensitive response [35,36,37,38,39,40]. However, the complex fabrication processes and mechanical instability limit the application of microstructures in flexible and portable gas sensors [41,42,43,44].

Herein, a simple hybrid solvent strategy is innovatively proposed to modulate the intrinsic microstructure of the sensitive layer in a fully stretchable OFET NO_2_ sensor. An active layer with nanoneedle-like microstructures is fabricated by blending the polymer poly[2,5-bis(2-decyltetradecyl)pyrrolo[3,4-c]pyrrole-1,4(2H,5H)-dione-alt-5,5-di(thiophen-2-yl)-2,2-(E)-2-(2-(thiophen-2-yl)vinyl)thiophene] (PDVT-10) with the elastomer polystyrene-block-poly(ethylene-random-butylene)-block-polystyrene (SEBS) in a binary solvent system. Unlike conventional rigid substrate-dependent methods, this strategy enables large-area solution processing without requiring additional lithography or imprinting steps. This study achieves a breakthrough in balancing electrical performance, mechanical stretchability, and gas-sensing capabilities.

## 2. Materials and Methods

### 2.1. Raw Materials

PDVT-10 was purchased from Ruixun Optoelectronic Material Ltd. (Shenzhen, China). And SEBSs (H1221, H1052, and H1062) were purchased from Asahi Kasei Co. (Tokyo, Japan). Among them, SEBS H1221 was used as a semiconductor film, SEBS H1062 was used as an elastomer substrate, and SEBS H1052 was used as a dielectric layer. Single-walled carbon nanotube (SWCNT) solution was purchased from Organic Chemistry Ltd., Chinese Academy of Sciences (Chengdu, China). The solvents o-xylene, toluene, acetone, anhydrous ethanol, n-hexane, and trichloromethane were purchased from Yuanli Chemical Ltd. (Tianjin, China). o-dichlorobenzene was purchased from Aladdin Biochemical Technology Ltd. (Shanghai, China). Gallium–indium alloy (Ga/In:40:60) was purchased from Hongfeng Weili Science and Technology Development Ltd. (Tianjin, China). SiO_2_/Si was purchased from the 46th Research Institute of China Electronics Technology Group Corporation (Tianjin, China). All reagents were used directly without further purification.

### 2.2. Preparation of Organic Semiconductor Polymer Films

The preparation process of stretchable organic semiconductor polymer films is shown in Figure S1. (1) Dissolve: A blended solution of conjugated polymer PDVT-10 and elastomer SEBS H1221 was prepared in a single organic solvent (the blending ratio was 70 wt%, i.e., mixed in a ratio of 70% SEBS to 30% PDVT-10 by weight), in which SEBS served to improve the semiconductor mechanical properties [45]. Dissolving was carried out by heating and stirring at 95 °C for 6 h. (2) Preparation of substrate: To obtain rigid substrates, SiO_2_/Si substrate was subjected to O_2_ plasma treatment and octadecyltrimethoxysilane (OTS) modification [46]. Then, the SiO_2_/Si substrate was sequentially ultrasonically cleaned with deionized water, acetone, and isopropanol for 10 min and blown dry using a nitrogen gas gun. O_2_ plasma treatment, i.e., oxygen plasma treatment, was carried out for 10 min (with an intensity of 80 mW). OTS modification was achieved by exposing the sample to OTS vapor overnight. The SiO_2_/Si substrate was cleaned again via ultrasonication with trichloromethane, n-hexane, and isopropanol sequentially for 10 min, and it was blown dry with a nitrogen gun. (3) Solution processing for film formation: The polymer blending solution was used to form a film on the substrate (on the OTS-treated SiO_2_/Si sheet) using the spin-coating or solution-shearing method. The spin-coating method [47] was carried out at 2000 rpm for 60 s. The solution-shearing method was carried out as follows [46]. In the shear-assisted phase separation process, the substrate was fixed on a movable platform (Zaber X-LHM200A-E03, Zaber Technologies Inc., Vancouver, BC, Canada), and the scraper was tilted at 15° with respect to the substrate. The mixed solution (4 μL) was dripped into the space between the substrate and the scraper, and the distance between the two was fixed to 150 μm. The platform was moved by a motor at different speeds to form the polymer solution. (4) Annealing: The obtained films were annealed in a vacuum oven at 160 °C for 50 min to complete the preparation of high-performance PDVT-10/SEBS organic semiconductor films.

### 2.3. Preparation of Organic Field-Effect Transistor Devices

The preparation process of a rigid device with a bottom-gate/top-contact structure is shown in Figure S2. (1) The rigid substrate was an OTS-treated SiO_2_/Si sheet. (2) The dielectric layer was SiO_2_. (3) The semiconductor layer was PDVT-10 70 wt% SEBS blended film M1–M6. The multicomponent system OSC was scratch-coated on the OTS-treated SiO_2_/Si sheet using the solution-shearing method, and it was annealed for 50 min in a vacuum oven at 160 °C. (4) The source/drain poles were a pair of parallel Au films (100 nm). The electrostatic lamination method was used to bond a gold film (pre-deposited via evaporation) to a semiconductor layer via electrostatic force, avoiding damage to the semiconductor film caused by direct metal evaporation. (5) The gate was a gallium–indium alloy. The prepared sample was placed on a slide coated with gallium–indium alloy. Finally, the preparation of the fully stretchable OFET device was completed, and the device structure is shown in Figure S3.

**Figure 1:**
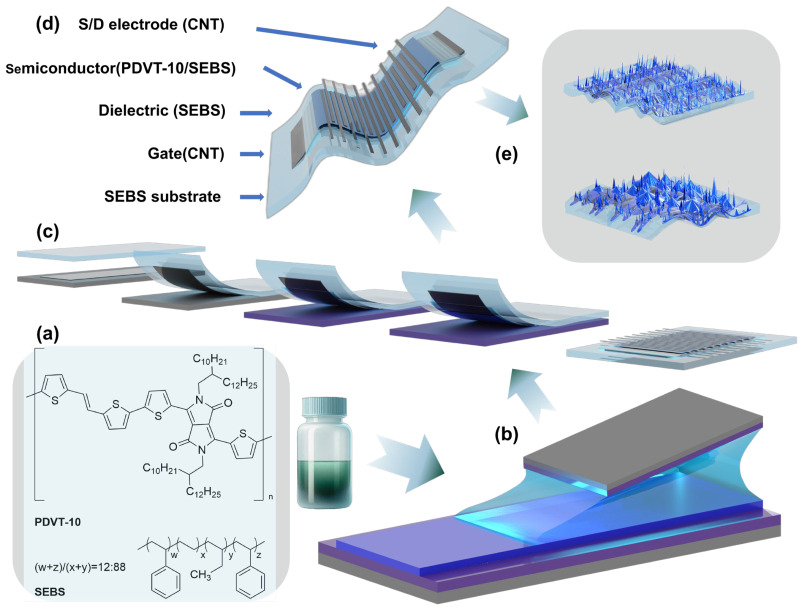
Design of the PDVT-10/SEBS OFET NO_2_ sensor. (**a**) Chemical structures of semiconducting polymer PDVT-10 and SEBS elastomer. (**b**) Schematic illustration of the solution-shearing process for the preparation of PDVT-10/SEBS films. (**c**) Schematic of the layer-by-layer transfer process for the preparation of a fully stretchable PDVT-10/SEBS OFET. (**d**) Schematic illustrating the PDVT-10/SEBS OFET NO_2_ sensor. (**e**) Schematic illustrating the intrinsic microstructure of the sensitive layer of the sensor.

The preparation process of a flexible device with a bottom-gate/top-contact structure is shown in Figure 1. (1) The flexible substrate was SEBS substrate. A solution of SEBS H1062 in solvent (250 mg/mL) was cast on a clean Si wafer and allowed to dry. (2) The gate comprised CNTs. A solution of SWCNTs was sprayed onto a clean Si wafer using a commercial spray gun. (3) The dielectric layer was SEBS film. A solution of SEBS H1052 (60 mg/mL in toluene) was spin-coated onto an OTS-treated Si substrate (following the same procedure used for the SiO_2_/Si substrate) at 1000 rmp/min. (4) The semiconductor layer was a PDVT-10 70 wt% SEBS blended film. The multicomponent system OSC was deposited onto the octadecyltrichlorosilane (OTS)-treated SiO_2_/Si substrate using the solution-shearing coating method and subsequently annealed at 160 °C in a vacuum oven for 50 min. (5) The source/drain electrodes were also CNTs. The Si substrate was covered with a designed mask plate using the inkjet printing method (Figure S4), and SWCNT solution was sprayed onto the OTS-treated Si substrate using a commercial spray gun. Finally, using the layer-by-layer transfer method (Figure S5), the preparation of fully stretchable OFET devices was completed, and the device structure is shown in Figure 1. At this time, the channel length/channel width (*L*/*W*) was 0.25, and the dielectric layer capacitance (*C*_i_) was 15 mF/m^2^.

### 2.4. Structural Characterization of Organic Semiconductor Polymer Films

GIXD tests were performed at the Shanghai Synchrotron Radiation Light Source. The wavelength of the X-rays was 0.97 Å, the energy was 12.7 keV, and the angle of incidence of the X-rays was 0.12°. The distance from the sample to the detector was approximately 400 mm. GIXD measurements were performed in a helium atmosphere to minimize air scattering [48]. The absorption intensity of the thin-film polymer was measured using a PE lambda 750 UV–Vis spectrometer (PerkinElmer, Waltham, MA, USA). X-ray photoelectron spectroscopy (XPS) was used to analyze the chemical composition of the blended films at different depths. Optical microscopy was used to observe the cracking of the blended films under strain. The elastic modulus of the films was measured by tensile testing [49]. The microscopic surface morphology of the blended films was characterized using atomic force microscopy (AFM). The overall surface morphology of the films was observed using scanning electron microscopy (SEM).

### 2.5. Performance Testing of Organic Field-Effect Transistor Devices

(1) Characterization of basic mechanical properties: Stress–strain curves were obtained using film tensile tests. An optical microscope was used to record the changes in surface morphology before and after the stretching of the films. (2) Characterization of basic electrical properties: The electrical properties of the stretchable OFETs and rigid OFETs were characterized in air using a semiconductor analyzer Keithley 4200 (Keithley, Cleveland, OH, USA), with a probe station. (3) Mechanical–electrical performance characterization: The mechanical–electrical performance of the flexible OFETs in the stretched state or after stretching was characterized in air using a semiconductor analyzer, Keithley 4200, with a probe station and a manual/electrical stretching device. (4) Characterization of gas-sensing performance: The gas-sensing performance of single gas-sensing components with the rigid and flexible organic field-effect transistors was characterized in a gas-sensing device using a semiconductor analyzer Keithley 2636B (Keithley, USA), which manifested as a change in electrical performance. In this experiment, synthetic air was used as the background gas to equilibrate the gases, and NO_2_, nitric oxide, ammonia, carbon monoxide, sulfur dioxide, trimethylamine, ethylene, acetone, methanol, and hydrogen sulfide gases were used as the analytical gases. As polymer semiconductors typically exhibit an extremely slow saturation trend at room temperature for NO_2_ [50], the steady-state and transient responses of the PDVT-10/SEBS OFET needed to be studied by limiting the pulse width of the analyzed gas [51]. Figure S5 shows a schematic diagram of the connection of the probe station to the Keithley-4200 or Keithley-2636B.

The test methods and parameter configurations for the performance test were as follows: (1) In the transfer characteristic test, the source–drain voltage *V*_DS_ was set to −60 V (for the rigid device) or −40 V (for the flexible device), the gate voltage *V*_G_ was set to 30~60 V, and the clamp current was set to 10 mV. (2) For gas testing, the pulse width was limited at NO_2_ (an exposure time of 1 min or 3 min and a total flow rate of 1000 mL/min). (3) In the steady-state gas sensitivity characterization, a D4’ sensor was used as a model device, and transfer curves were recorded at different gas concentrations (0 ppm, 1 ppm, 2 ppm, 3 ppm, 4 ppm, 5 ppm, 10 ppm, 20 ppm, 30 ppm, 40 ppm, and 50 ppm). (i) The variation in gas responsivity with gate pressure modulation under different NO_2_ gas concentration conditions was calculated and plotted using the equation *R* = (*I*_DS_ − *I*_0_) × 100%. (ii) The average sensitivity was determined based on the slope of the linear plot of the response at different concentrations. (4) In the transient gas-sensitive characterization study using the D4’ sensor as the model device, the *I*-*t* curve of the real-time response to a 10 ppm gas concentration was measured with a constant current and voltage output after the device was turned on. In order to test the reliability of the variation in the current response, the real-time gas response *I*-*t* curve of D4’ was recorded for 100 cycles by repeatedly applying the gate voltage of the stepped output. The stepped output gate voltage *V*_G_ comprised the turn-on voltage *V*_on_ and the maximum gate voltage, respectively, in 100 consecutive cycle tests. (5) During the investigation of the enhancement mechanism of the gas-sensitive performance of the NO_2_ sensor, the tests included the following: (i) transfer curves under different NO_2_ gas concentration conditions; (ii) change curves of gas responsiveness with the regulation of gate pressure; (iii) the variation in gas responsivity with the NO_2_ gas concentration under the condition of *V*_G_ = *V*_on_; and (iv) transient studies, i.e., real-time source–drain current $I_{DS}$ variation with the gas concentration. (6) In the reproducibility test, the experiment was conducted with 10 ppm of NO_2_ used as the analyzed gas, with a fixed exposure time of about 3 s, and the data showed a change in *I*_DS_ for about 6 s, with a cycle period of 220 s. According to the saturation region and subthreshold region of the D3 and D4 devices, the settings of *V*_DS_ = −40 V and *V*_G_ = −15 V were used for the single-solvent system D3, and *V*_G_ = −4 V was used for the mixed-solvent system D4; then, the changes in the channel current *I*_DS_ were measured.

## 3. Results and Discussion

### 3.1. Device Design of Stretchable Organic Semiconductors Based on Multicomponent Systems

The key factors in achieving better sensing performance of high-mobility stretchable OSCs with multicomponent systems capable of nanolimiting effects are (1) efficient charge transport, (2) efficient mechanical stretching, (3) efficient gas absorption, and (4) efficient gas-selective responses. Based on the phase separation behavior of rigid/elastic polymer blends, we chose the rigid donor–acceptor polymer PDVT-10, as well as the thermoplastic elastomer polymer SEBS. The chemical structures of PDVT-10 and SEBS are shown in Figure 1a. In order to optimize the preparation conditions of fully stretchable OSCs for multicomponent systems, the average mobility *μ*_eve_ and the highest mobility *μ*_max_ of the OSCs prepared using the spin-coating and solution-shearing methods were compared. As shown in Figure S7, the OSCs obtained using the solution-shearing method had a stronger charge transport ability. Figure 1b presents a schematic diagram of the process of producing PDVT-10/SEBS blended films on Si/SiO_2_ substrate using the solution-shearing method. On this basis, the layer-by-layer transfer method was used, as shown in Figure 1c, to prepare fully stretchable OFET devices with a multicomponent system, and the device structure is shown in Figure 1d. In the present study, a simple mixed-solvent strategy was innovatively used for the intrinsic microstructure design of the sensitive layer, and the microstructure is schematically shown in Figure 1e. The structure facilitated the enhancement of both the charge transfer efficiency of the fully stretchable OFET device and the NO_2_ gas response of the OFET flexible sensor.

### 3.2. Structural Characterization of Stretchable Organic Semiconductors Based on Multicomponent Systems

The charge transport properties of the thin films with o-xylene as the solvent (M1), p-xylene as the solvent (M2), o-xylene as the solvent (M3), a mixture of o-xylene and o-xylene in a ratio of 7:3 as the solvent (M4), a mixture of o-xylene and o-xylene in a ratio of 3:7 as the solvent (M5), and a mixture of o-xylene and o-xylene in a ratio of 1:1 as the solvent (M6) were investigated. Obtained using the control variable method, the transfer curves and optical microscope images of the thin-film devices under different solution-shearing temperatures are shown in Figures S8–S13. Additionally, the test results obtained under different solution-shearing speeds are shown in Figure S14. The device mobility of the single-solvent system films (M1, M2, and M3) and the mixed-solvent system films (M4, M5, and M6) increased and then decreased with the increase in the solution-shearing temperature, and it increased and then decreased with the increase in the solution-shearing speed. The solution-shearing temperatures with the highest mobility were 105 °C, 75 °C, and 80 °C for M1, M2, and M3, respectively, and 85 °C, 95 °C, and 90 °C for M4, M5, and M6, respectively.

The thickness and uniformity of the films can be reflected by the color change in the OM images in Figures S8–S13, where the films change from thin to thick, and the color changes from blue to red. According to the AFM characterization, the thicknesses of the M1, M3, and M4 films at the solution-shearing temperature of 85 °C were about 70 nm, 110 nm, and 110 nm, respectively, as shown in Figure S15. The surface roughness of the M3 and M4 films varied with the shear temperature versus scanning area, as shown in Tables S1 and S2. On the one hand, the same set of organic solvent systems was compared. The film thickness tended to increase with an increasing shear temperature (Figures S8–S13) and decrease with an increasing shear rate (Figure S14). The average roughness Ra showed a decreasing and then increasing trend with an increasing shear temperature (Tables S1 and S2). In addition, Ra varied in a scanning area-dependent manner. The large region (16.6 μm × 16.6 μm) mirrored the macroscopic phase separation behavior, and its Ra was more sensitive to temperature fluctuations. The small region (1.83 μm × 1.83 μm) reflected the nanoscale homogeneity, and its Ra had less data fluctuations. On the other hand, different groups of organic solvent systems were compared. Overall, the degree of uniformity was in the order of M1 > M5 > M6 > M4 > M3 > M2, and the degree of thickness was in the order of M2 > M3 > M4 > M6 > M5 > M1. In addition, M1 had high mobility but did not easily form a film using the solution-shearing method, while M3 and M4 exhibited high carrier mobility, excellent film-forming capabilities, a high fault tolerance, and a greater ease of transfer during the preparation of flexible devices. In the subsequent work, M3 with a solution-shearing temperature of 80 °C and M4 with a solution-shearing temperature of 85 °C were chosen as the models for the study of thin films in single- and mixed-solvent systems, respectively.

**Figure 2:**
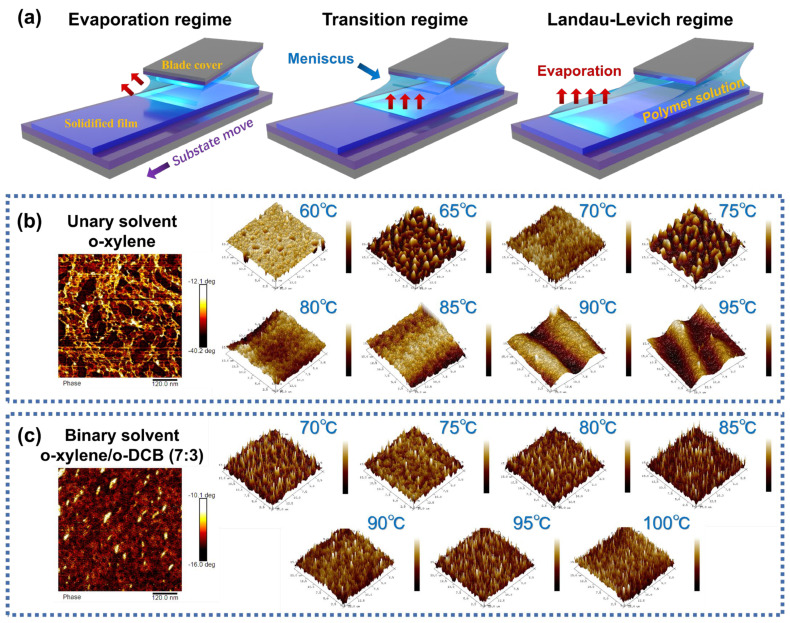
Morphological characterization of the PDVT-10/SEBS films. (**a**) The solution-shearing-assisted film formation mechanism. (**b**) The 2D AFM phase diagrams (left) and 3D AFM height diagrams (right) of M3. (**c**) The 2D AFM phase diagrams (left) and 3D AFM height diagrams (right) of M4.

During the formation of the solution film, the morphology of PDVT-10/SEBS OSC films can be adjusted via capillary force, shear force, and Marangoni flow through the dynamic modulation effect on the meniscus shape [52]. This affects parameters such as film thickness and roughness. The results are consistent with the three shear mechanisms summarized by researchers, namely the evaporation, transition, and Landau–Levich (LL) regimes, as shown in Figure 2a. These three regimes, in turn, correspond to low, medium, and high shear velocities and high, medium, and low shear temperatures, respectively. Among them, the M4 transition system (with a shear speed of 0.37 mm/s and a shear temperature of about 85 °C) had a higher film mobility (1.23 cm^2^ V^−1^ s^−1^), a smaller thickness (about 100 nm), and a lower roughness (Ra = 0.55 nm).

Next, 3D AFM images were used to characterize the effects of different solution-shearing temperatures and the choice of solvent system on the surface morphology of the films, as shown in Figure 2b,c. It could be observed that the thin film exhibited intrinsic microstructures induced by phase separation, primarily manifested as nanoscale-distributed “protrusion” features. By modifying the organic solvent system, the morphology, size, and distribution of these nanostructures could be effectively tuned. For instance, island-like protrusions formed in the single-solvent system (M3), while needle-like protrusions emerged in the binary mixed-solvent system (M4). Notably, at the micrometer scale, M3 exhibited pore-like, droplet-like, island-like, and wavy structures, whereas M4 maintained its needle-like microstructure without significant alteration. To further compare the microstructures on the surfaces of the stretchable multicomponent system films M1, M3, and M4, photographed AFM height maps in different scale ranges are shown in Figures S16 and S17. The average roughness of these films is summarized in Table S3. AFM phase images in different scale ranges are presented in Figure S18, with the images in the 608 nm × 608 nm scale ranges specifically shown in Figure 2b (left) and Figure 2c (left). A comparative analysis revealed that the surface protrusions on the M4 films (prepared using binary mixed-solvent systems) exhibited a more discrete distribution, whereas the M1 and M3 films (prepared using single-solvent systems) were more aggregated. It was hypothesized that the distinct properties of the two solvents in the mixed system induced an asynchronous and structurally dissimilar phase separation of PDVT-10 in SEBS, which led to the different distributions of the microstructures on the surfaces of the films.

In addition, the amorphous halo properties observed in the GIWAXS measurements (Figure S19) are evidence that the incorporation of SEBS contributed to the improvement of the mechanical properties of the polymer blends. The intensity of the absorption peaks at 765 nm in the UV–Vis absorption spectra curve (Figure S20) indicates the degree of aggregation of PDVT-10 in the films, and it could be seen that M4 > M3 > M1. The ratio of the S 2p characteristic peaks to the C 1s characteristic peaks in the X-ray photoelectron spectroscopy (XPS) can qualitatively reflect the changes in the distribution of the components of PDVT-10 and SEBS in the film along the thickness direction (Figure S21). Specifically, M1 formed a “sandwich” structure; i.e., PDVT-10 was concentrated at the top and bottom of the film and less in the middle. Correspondingly, M3 had a tendency to enrich PDVT-10 toward the surface of the film more than M1, and M4 tended to be distributed toward the middle and upper layers more clearly. The OM images (Figure S23) show that the SEBS-undoped film had obvious cracks after 100 cycles of cyclic stretching at 50% strain, while no cracks were observed in the two multicomponent systems M3 and M4 doped with SEBS. Thus, the nanoconfinement effect between PDVT-10 and SEBS is highly desirable, featuring low crystallinity, a high aggregation degree, and unique nanofiber networks. Simultaneously, mixed-solvent processing enables the formation of intrinsic microstructures with a nanoscale spatial distribution.

### 3.3. Force–Electric Property Balance of Stretchable Organic Field-Effect Transistors

**Figure 3:**
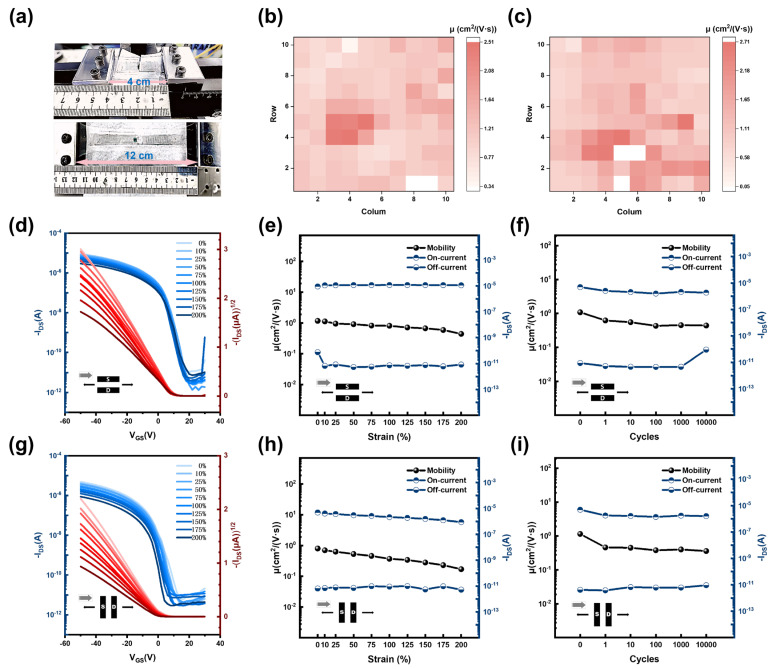
Mechanical–electrical properties of fully stretchable OFETs. (**a**) Physical images at 0% and 200% strain. (**b**) Mobility distribution in fully stretchable D3 OFET arrays. (**c**) Mobility distribution in fully stretchable D4 OFET arrays. (**d**) Transfer curves of the D4 device under different strains (TD⟂CTD). (**e**) Changes in on/off current and mobility of the D4 device under different strains (TD⟂CTD). (**f**) Changes in on/off current and mobility of the D4 device after multiple stretch–release cycles (up to 10000 cycles) at 100% strain (TD⟂CTD). (**g**) Transfer curves of the D4 device under different strains (TD‖CTD). (**h**) Changes in on/off current and mobility of the D4 device under different strains (TD‖CTD). (**i**) Changes in on/off current and mobility of the D4 device after multiple stretch–release cycles (up to 10000 cycles) at 100% strain (TD‖CTD).

The fully stretchable OFET devices D3 and D4, based on PDVT-10/SEBS, were prepared using the single-solvent system (o-xylene) M3 and the mixed-solvent system (o-xylene–o-dichlorobenzene = 7:3) M4 as the OSC films. The fully stretchable OFET devices had remarkable mechanical tensile properties up to a strain of 578%, as shown in Figure S24. Photographs of them in the initial state and at 200% tensile strain are shown in Figure 3a. In addition, the basic electrical properties of the devices were characterized in air, as shown in Figure S25. At 0% strain, the maximum mobility of D3 was 2.51 cm^2^ V^−1^ s^−1^, and that of D4 was 2.71 cm^2^ V^−1^ s^−1^. The mobility distributions in the fully stretchable OFET arrays (10 × 10) are shown in Figure 3b,c. The average mobility of D3 and D4 was 1.17 cm^2^ V^−1^ s^−1^ and 1.30 cm^2^ V^−1^ s^−1^, with a standard deviation of 0.43 and 0.54, respectively. This shows that the homogeneity of M3, prepared with a single-solvent system, is better, and the charge transport performance of M4, prepared with a mixed-solvent system, is better.

In order to characterize the mechanical–electrical performance of the fully stretchable OFET devices in the stretched state, measurements were conducted at different strains (0%, 10%, 25%, 50%, 75%, 100%, 125%, 150%, 175%, and 200%), with the stretch–tensile direction (TD) parallel or perpendicular to the charge transport direction (CTD). The transfer curves of D3 and its on/off currents and mobility variations are shown in Figure S26. The transfer curves of D4 and its on/off currents and mobility variations are shown in Figure 3d,e (TD⟂CTD) and Figure 3g,h (TD‖CTD). At 100% strain, the maximum mobilities of D3 and D4 were 1.21 cm^2^ V^−1^ s^−1^ and 0.89 cm^2^ V^−1^ s^−1^, respectively. Among them, the carrier mobility of D4 was reduced by 29.91%. At 200% strain, the mobilities of D3 and D4 were reduced by 56.97% on average. Among them, the devices were more strain-adaptable at TD⟂CTD than at TD‖CTD, as evidenced by a slower decrease in the increasing mobility. In addition, the durability of the D3 and D4 devices under repetitive strain is shown in Figures S27 and S28. After 1000 stretch–release cycles at 100% strain under TD‖CTD or TD⟂CTD, D3 and D4 still maintained normal electrical performance, with an average mobility reduction of 46.71%. Among them, D4 was supplemented with measured transfer curves after 10,000 cycles, as shown in Figure 3f,i. The test data showed that D4 exhibited excellent cycling durability (95% retention of mobility in the 100–10,000-cycle interval) after the first cycle of mobility decline.

Further, the effects of TD, CTD, and the solution-shearing direction (SSD) on the mechanical–electrical properties of the devices were explored. The mechanical–electrical properties are shown in Figure S29, and the durability under repetitive strains is shown in Figure S30. By comparing the initial mobility under 0% strain, it was found that D4 (TD‖SSD, TD⟂CTD) > D4 (TD⟂SSD, TD‖CTD) > D4 (TD‖SSD, TD‖CTD) > D4 (TD⟂SSD, TD⟂CTD). The test results show that the highest initial mobility (at 0% strain) was obtained for the device when SSD‖CTD. Comparing the degree of mobility degradation under the same strain condition, it was found that D4 (TD⟂SSD, TD⟂CTD) < D4 (TD‖SSD, TD⟂CTD) < D4 (TD‖SSD, TD‖CTD) < D4 (TD⟂SSD, TD‖CTD). It could be observed that satisfying the TD⟂CTD condition helped to achieve better mechanical–electrical performance of the device. On this basis, satisfying the SSD⟂CTD condition had a more significant effect on the optimization of the mechanical–electrical performance. In addition, Figure S31 explains the strain adaptation mechanism of the stretchable OSC based on the multicomponent system.

### 3.4. Stretchable Organic Field-Effect Transistors for Gas-Sensing Applications

The relevant bias connections for evaluating the NO_2_ gas-sensing performance of the PDVT-10/SEBS OFET devices are shown in Figure S32. The devices that were tested included a fully stretchable OFET sensor, D3, prepared under single-solvent conditions (o-xylene); a fully stretchable OFET sensor, D4, prepared under mixed-solvent conditions (7:3 ratio mix of o-xylene and o-dichlorobenzene); and their rigid OFET sensors, D3’ and D4’. As polymer semiconductors typically exhibit an extremely slow saturation trend at room temperature for NO_2_ [50], the study of the steady state and transient states of the PDVT-10/SEBS OFET needed to be limited to the pulse width of the analyzed gas [51].

While limiting the pulse width of the analyzed gas to 3 min, the transfer curves of D4’ under different gas concentration conditions and the change curves of gas responsivity with gate voltage modulation were recorded, and the average sensitivity was calculated based on the slopes of the linear plots of the responses at different concentrations (Figure S33). The responsivity was calculated using *R* = (*I*_DS_ − *I*_0_) × 100%, and it increased from 6.8 × 10^3^ % at 1 ppm to 3.1 × 10^7^ % at 50 ppm. The average sensitivity of the rigid device was approximately 6.0 × 10^5^ %/ppm, with data calculated from the current values of the transfer characteristic curve in the subthreshold region. In contrast, the gas-sensing performance of D4’ for other small toxic gas molecules is shown in Figures S34 and S35. It could be observed that the OFET sensors of the multicomponent system with stretchable OSCs had an ultra-high response only to NO_2_, and the devices showed the maximum responsiveness in the subthreshold region (*V*_G_ ≈ *V*_on_). That is, the devices demonstrated remarkable response signal amplification in the subthreshold region. Furthermore, the real-time gas response *I*-*t* curve of D4’ was tested, and the specific parameter settings are shown in the experimental section. A comparison of Figures S36 and S37 revealed that the transient variation in the current was significant when NO_2_ was used as the analyzed gas. A comparison of Figures S38 and S39 revealed that the response current changed significantly at *V*_on_, especially for NO_2_ gas. Thus, in the subthreshold region (*V*_G_ ≈ *V*_on_), using the source–drain current of the device after it was turned on as the response parameter, the OFET sensor exhibited selectivity, as shown in Figure S40. Specifically, the D4’ sensor showed the highest response value (92,714.64%) for NO_2_ when the analyzed gas had a concentration of 50 ppm, while the value for the other gases did not exceed 100%.

**Figure 4:**
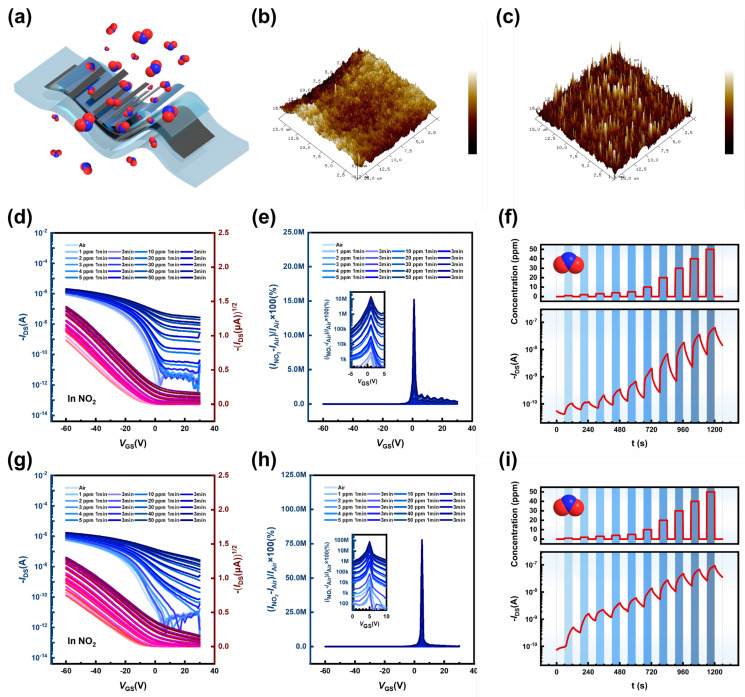
Sensing performance of the fully stretchable sensor toward various concentrations of NO_2_. (**a**) Schematic illustration of gas molecules’ interaction process in the fully stretchable PDVT-10/SEBS OFET device. (**b**) AFM topography images of M3. (**c**) AFM topography images of M4. (**d**) Transfer characteristics of the D3 device. (**e**) Variation in gas responsivity with gate voltage of the D3 device. (**f**) Transient analysis of the D3 device. (**g**) Transfer characteristics of the D4 device. (**h**) Variation in gas responsivity with gate voltage of the D4 device. (**i**) Transient analysis of the D4 device.

In order to investigate the enhancement mechanism of the gas-sensitive performance of stretchable OSCs in multicomponent systems, target gas-sensing tests were performed on rigid OFET devices (D3’ and D4’) and flexible OFET devices (D3 and D4), with the specific parameter settings described in the experimental section. Figures S41–S44 show the transfer curves with different NO_2_ gas concentration conditions, gas responsivity change curves with gate voltage modulation, gas responsivity changes with the gas concentration variation, and real-time source–drain current variations with the gas concentration obtained for the four groups of devices. The interaction of the gas molecules is shown in Figure 4a. The AFM images of the intrinsic microstructures of M3 and M4 are shown in Figure 4b,c. When the pulse width of NO_2_ was 1 min, the maximum responsivity of D3’ and D4’ to 50 ppm NO_2_ was over 2M% and 3.5M%, respectively, and that of D3 and D4 was over 11.7M% and 67.9M%, respectively. When the pulse width of NO_2_ was 3 min, the maximum response of the D3 and D4 devices to 50 ppm NO_2_ reached up to 15.2M% and 77.9M%, respectively. Compared to the D3 device (Figure 4d–f), the D4 device (Figure 4g–i) had a more pronounced response at low NO_2_ concentrations. Additionally, Figure S44 shows a remarkably high average sensitivity of 1.4 × 10^6^ %/ppm. As the NO_2_ concentration increased, the increasing trend of the D4 threshold voltage became more clear. This phenomenon was also verified by the real-time responsivity in Figure S45. Therefore, the fully stretchable devices D4 and D4’ prepared using the mixed-solvent strategy were more sensitive to NO_2_. A possible reason for this is that the nanoscale distribution on the sensitive layer of the devices was finer and more dispersed, which is favorable for the effective diffusion and interaction of NO_2_ with the active layer. As shown in Table S4, the proposed device demonstrates superior performance compared to existing devices of the same type.

The sensitive layer of the fully stretchable OFET sensor exhibited excellent operational stability (cyclic test and bias stability) and environmental stability (air, light, temperature, and humidity stability), as shown in Figures S46–S47. No significant performance degradation was observed in 100 randomized tests conducted over a one-month period. To explore the reproducibility of the sensing performance of the fully stretchable sensors, D3 and D4 were subjected to three exposure and recovery cycles (Figure S48). Additionally, after a number of consecutive 5 h exposure and recovery cycles, another eight exposure and recovery cycles were repeated (Figure S49). The average responsivity of D3 and D4 increased slightly, with growth rates of 5.45% and 11.66%, respectively. In addition, the fully stretchable OFET sensor exhibited excellent response and recovery behaviors (Figure S50). During the D4 response process, *I*_DS_ needed only 1.93 s to increase to 70% of its minimum initial current value.

To analyze the gas-sensing mechanism of the stretchable OFET sensor for multicomponent systems, from the device perspective, the current change and response signal were caused by the interfacial charge contention in the conductive channel after the oxidizing gas NO_2_ was adsorbed into the p-type PDVT-10/SEBS OSC. From a molecular point of view, the electrophilic group (single-bonded oxygen atom in the O=N-O structure) of NO_2_ and the electron-donating group (TVT) of PDVT-10 induced an increase in the hole concentration in the OSC layer through dipole interactions, and the unpaired electrons in the nitrogen atoms of the NO_2_ molecules enhanced its electron-trapping ability. Among them, sensing layer optimization is the key to coupling these mechanisms. Due to the solvent system modulation, the organic semiconductors formed thinner sensing functional layers, a finer PDVT-10 nanofiber mesh, and more dispersed intrinsic microstructures. The surface of the sensitive layer produced in the binary mixed-solvent system had nanoscale-distributed needle-like projections, as opposed to the island-like projections in the single-solvent system. This intrinsic microstructure is hypothesized to enrich gas molecules via capillary action [53,54,55,56], effectively enhancing the gas-sensing performance.

## 4. Conclusions

In summary, a straightforward hybrid solvent strategy to regulate the macrostructures of organic semiconductor layers was described herein for the construction of fully stretchable OFET NO_2_ sensors based on PDVT-10/SEBS. OFETs prepared via the binary mixed-solvent system (o-xylene–o-dichlorobenzene at about 7:3) showed nanoneedle-like raised microstructures. On the one hand, this intrinsic microstructure provided the OFETs with excellent mechanical and electrical properties. The maximum mobility of the device was 2.71 cm^2^ V^−1^ s^−1^, and the switching current ratio was typically higher than 10^6^. Under 100% strain conditions, mobility was reduced by 29.91%. On the other hand, outstanding NO_2_-sensing performance was achieved through the capillary action of the needle-like intrinsic microstructure, which enriched the gas molecules and enhanced their interaction with the sensitive layer. The device showed a high response to NO_2_ (up to 77.9 × 10^6^% at 3 min), high sensitivity (1.4 × 10^6^ %/ppm), good selectivity (<100% response to nine interferences), and good stability (1 month). Therefore, this study highlights the role of hybrid solvent systems in modulating film morphology and device performance, providing a fundamental platform for next-generation smart sensing technologies.

## Data Availability

Data is contained within the article or Supplementary Materials.

## Supplementary Materials

The following supporting information can be downloaded at https://www.mdpi.com/article/10.3390/nano15120922/s1, Section S1: Experimental section. (References [57,58,59,60,61] are cited in Supplementary Materials).
