# Supplementary material for: Binary Solvent Engineering Modulates the Microstructure of Stretchable Organic Field-Effect Transistors for Highly Sensitive NO2 Sensing
Source: Nanomaterials (Basel). 2025 Jun 13;15(12):922. doi: 10.3390/nano15120922 (PMC12195893; doi:10.3390/nano15120922)
Supplement: Supplementary file 1 [file nanomaterials-15-00922-s001.zip › nanomaterials-3666307-supplementary.pdf]

Supplementary Materials

Binary Solvent Engineering Modulates the Microstructure of Stretchable Organic Field-Effect Transistors for Highly Sensitive NO_2 Sensing

Xiao Jiang, Jiaqi Zeng, Linxuan Zhang, Zhen Zhang and Rongjiao Zhu*

Key Laboratory of Organic Integrated Circuits, Ministry of Education, Tianjin Key Laboratory of Molecular Optoelectronic Sciences, Department of Chemistry, School of Science, Tianjin University, 300072, China

S1 Experimental section

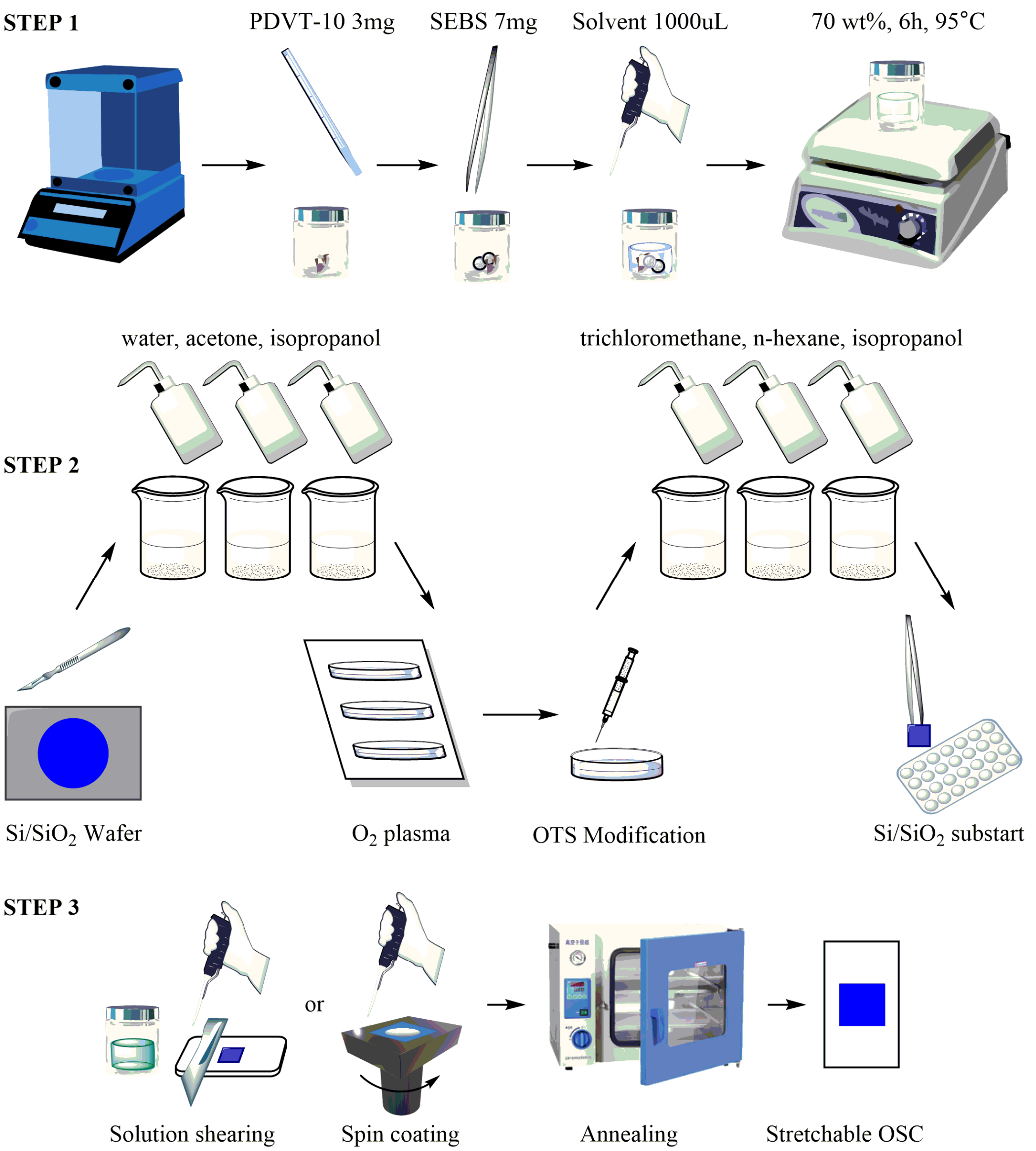

Figure S1 Preparation of organic semiconductor materials.

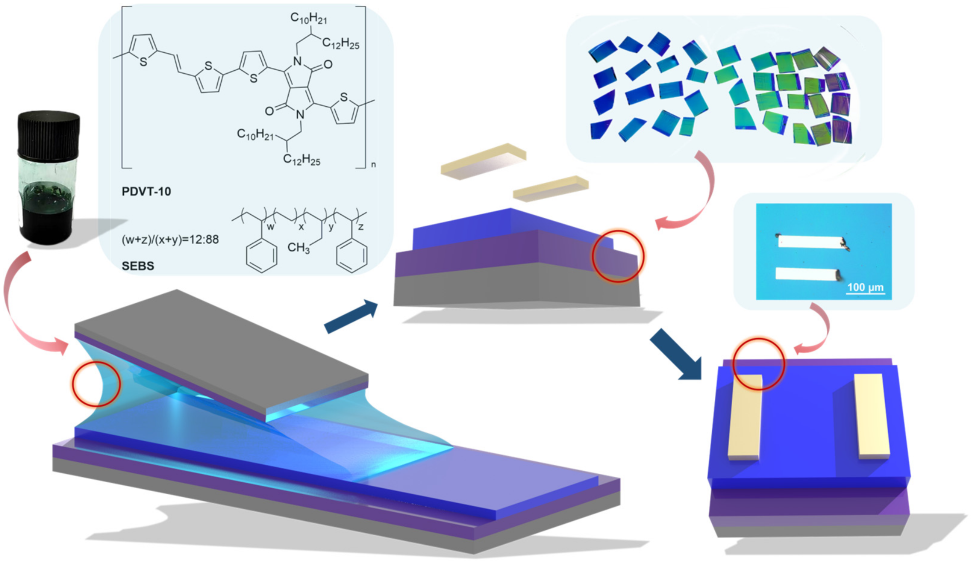

Figure S2 Preparation of organic field effect transistors.

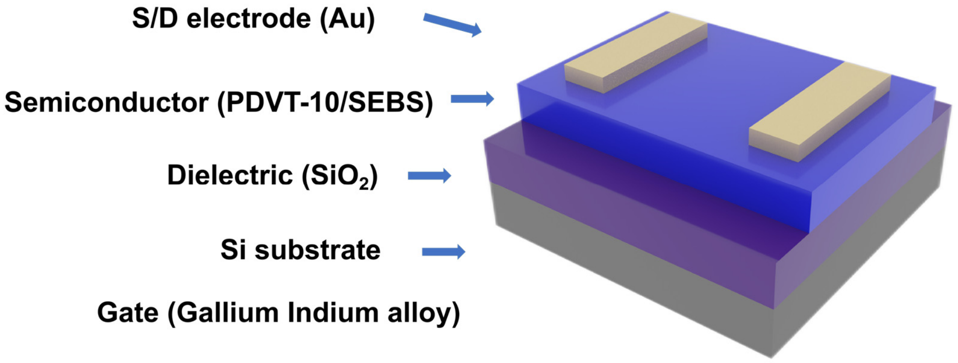

Figure S3 Rigid (silicon substrate) organic field-effect transistor.

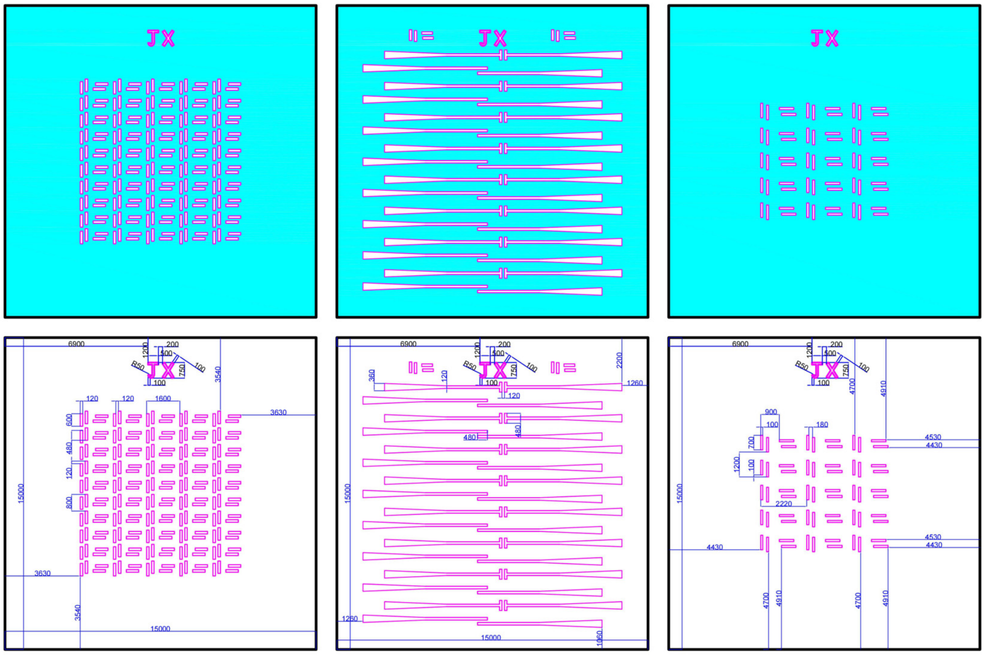

Figure S4 Inkjet printing technology and mask design.

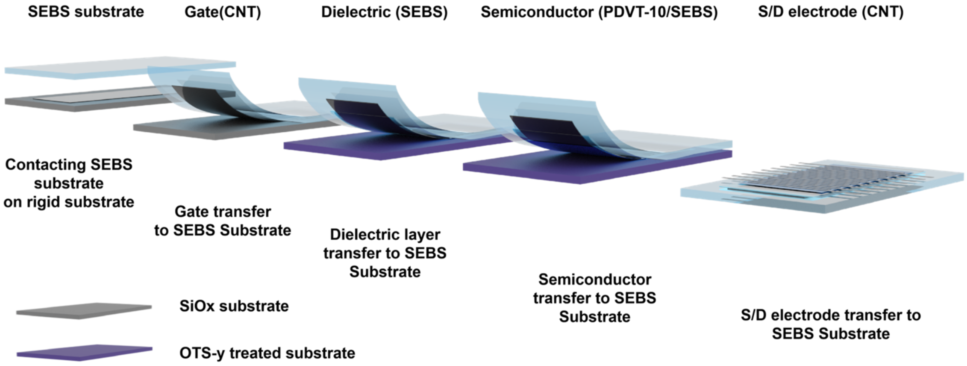

Figure S5 Schematic diagram of the fabrication of the fully stretchable OFETs (layer-by-layer transfer method).

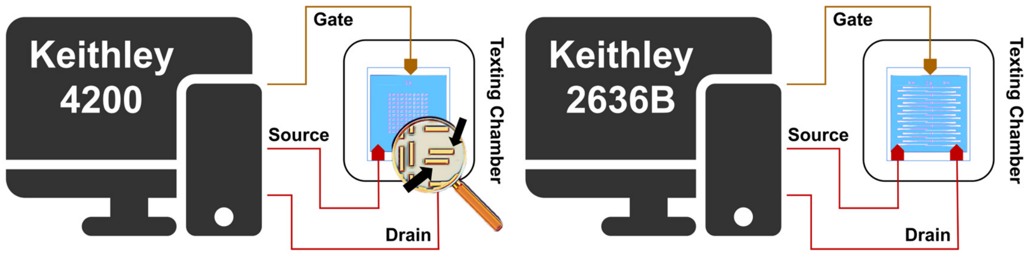

Figure S6 Schematic diagram of the probe stage connected to a Keithley-4200 (left) or a Keithley-2636B (right).

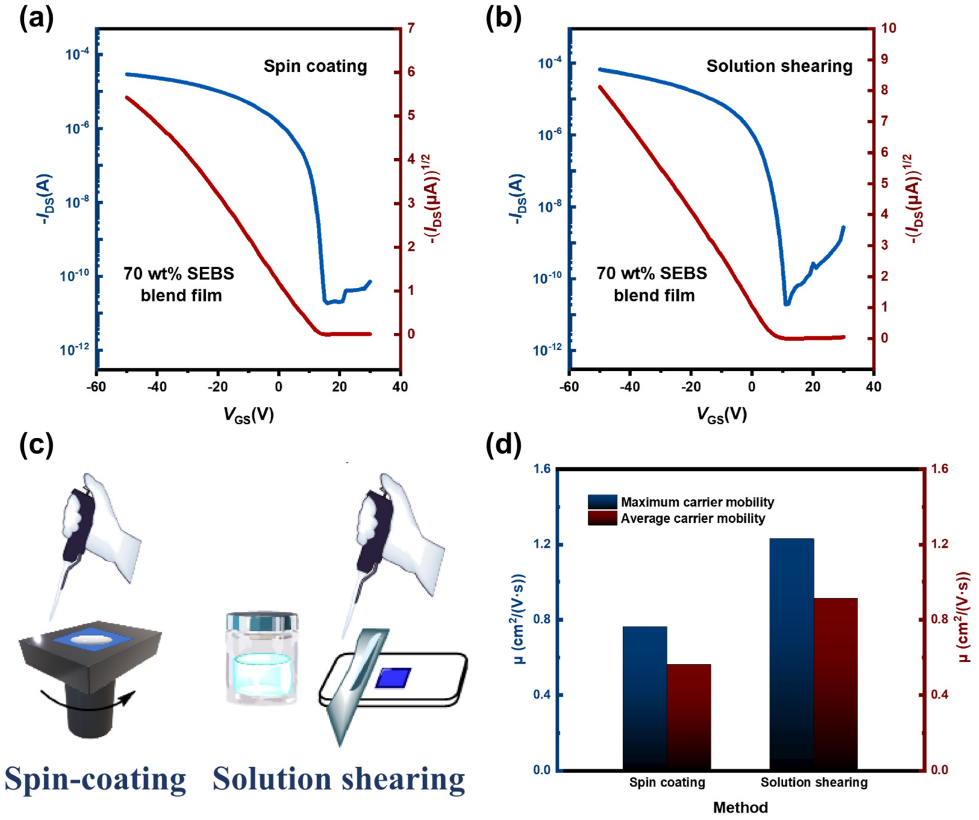

Figure S7 Spin-coating method and solution shear method.

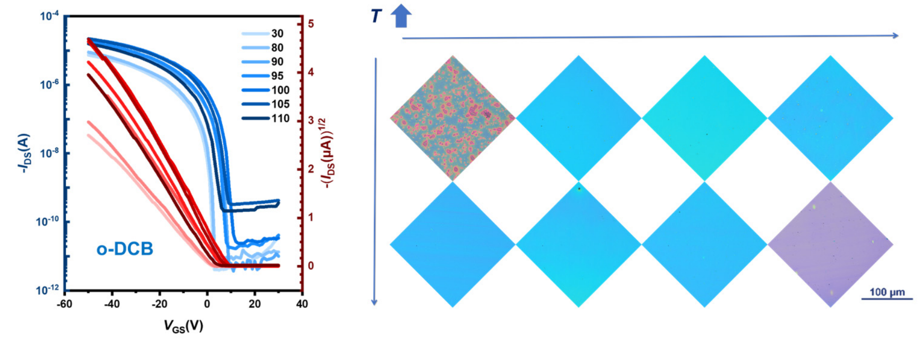

Figure S8 Transfer curves and optical microscope images of thin film M1 under different solution shear temperature conditions.

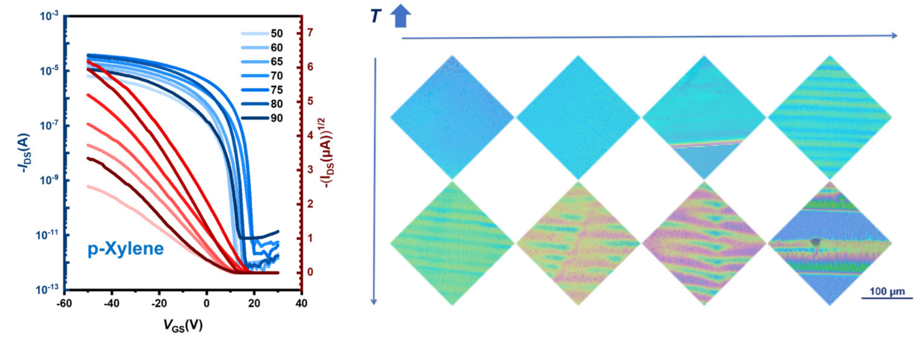

Figure S9 Transfer curves and optical microscope images of thin film M2 under different solution shear temperature conditions.

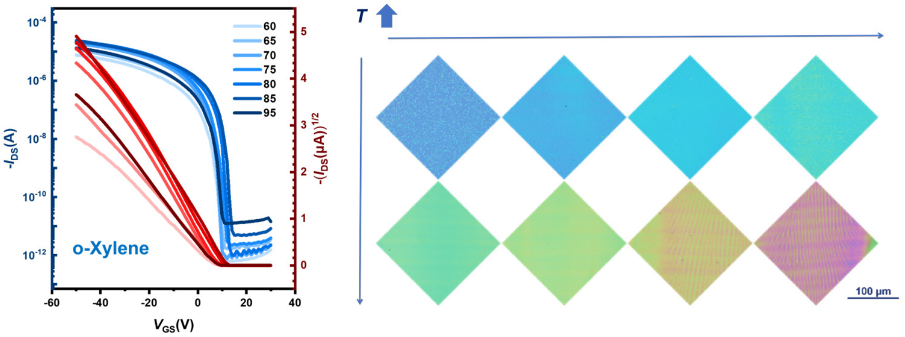

Figure S10 Transfer curves and optical microscope images of thin film M3 under different solution shear temperature conditions.

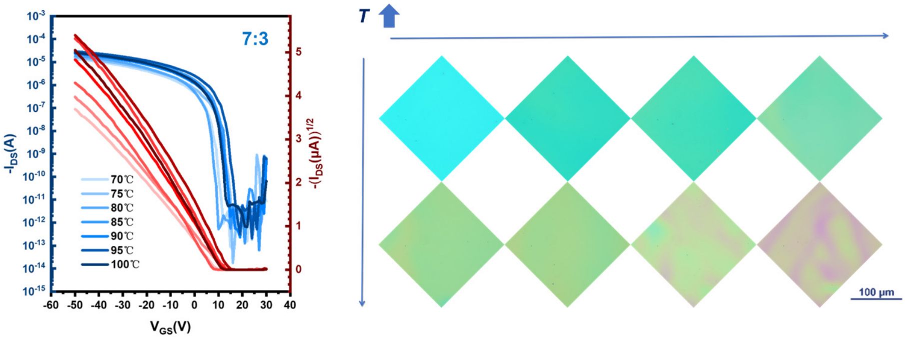

Figure S11 Transfer curves and optical microscope images of thin film M4 under different solution shear temperature conditions.

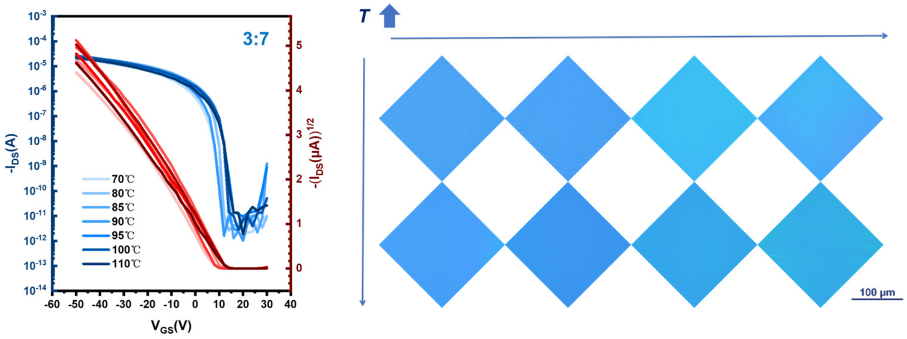

Figure S12 Transfer curves and optical microscope images of thin film M5 under different solution shear temperature conditions.

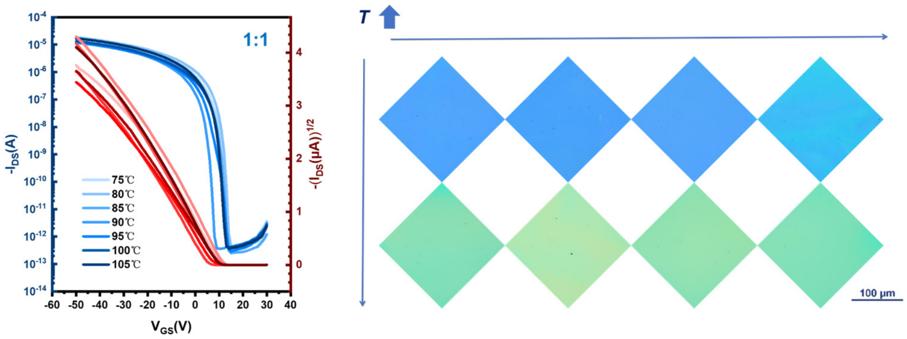

Figure S13 Transfer curves and optical microscope images of thin film M6 under different solution shear temperature conditions.

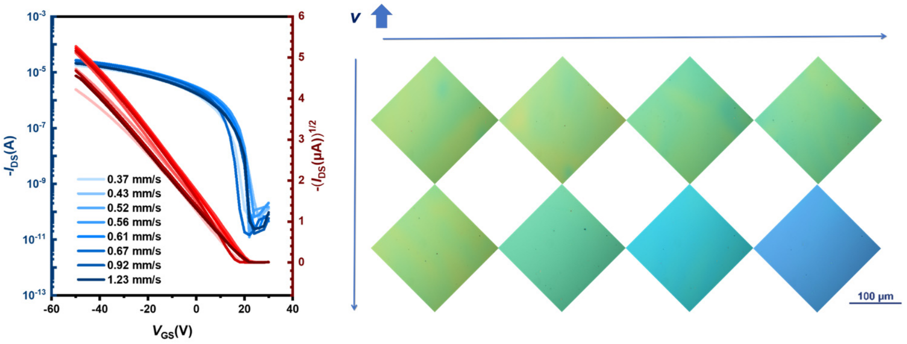

Figure S14 Transfer curves and optical microscope images of thin film M4 under different solution shear rate conditions.

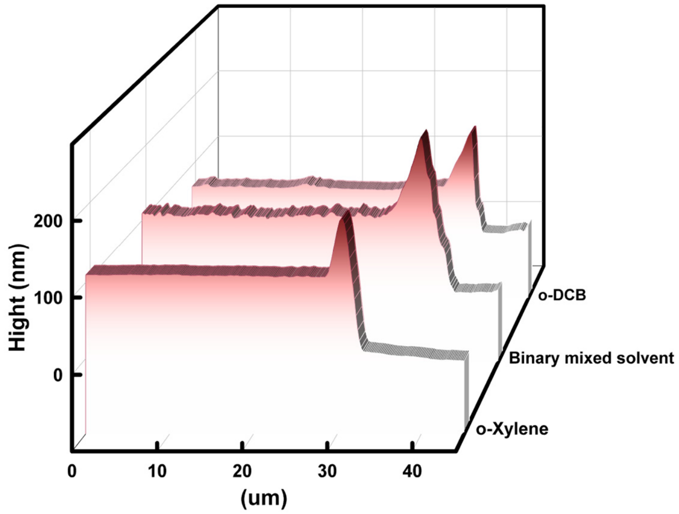

Figure S15 Solution film thickness (data taken from AFM characterisation).

Table S1 Variation of M3 surface roughness with shear temperature and scanning area for single solvent system.

Scan size (μm×μm) \ Ra (nm) \ T (°C)	60°C	65°C	70°C	75°C	80°C	85°C	90°C	95°C
16.6×16.6	2.02	12.7	1.21	4.43	1.40	2.52	5.37	7.38
5.51×5.51	3.34	11.9	0.47	3.45	0.90	1.04	0.99	2.54
1.83×1.83	1.06	8.90	0.42	0.81	0.66	0.55	0.76	1.44

Table S2 Variation of M4 surface roughness with shear temperature and scanning area for binary mixed solvent system.

Scan size (μm×μm) \ Ra (nm) \ T (°C)	70°C	75°C	80°C	85°C	90°C	95°C	100°C
16.6×16.6	2.53	1.11	1.15	0.952	0.687	1.57	1.92
5.51×5.51	3.28	0.910	1.02	0.735	0.544	1.47	1.68
1.83×1.83	1.83	0.90	0.69	0.56	0.49	1.06	1.14

Table S3 Variation of surface roughness with scanning area for different solvent systems.

Scan size (μm×μm) \ Ra (nm) \ Type	M1	M3	M4
50.0×50.0	2.05	7.74	3.33
16.6×16.6	1.22	1.16	0.89
5.51×5.51	1.18	1.15	0.92

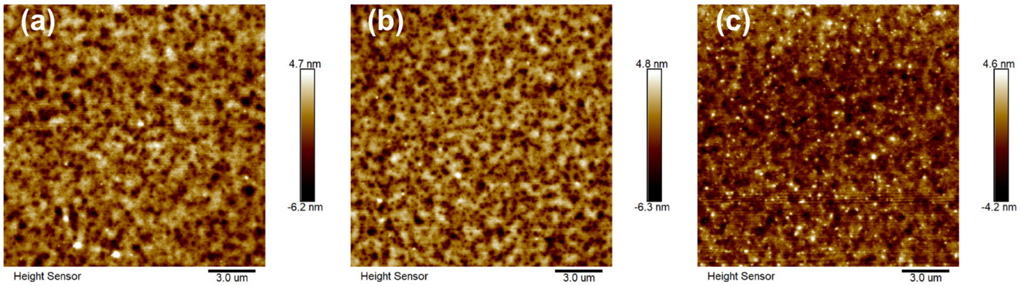

Figure S16 AFM height maps (16 μm×16 um) of films (a) M1, (b) M3 and (c) M4.

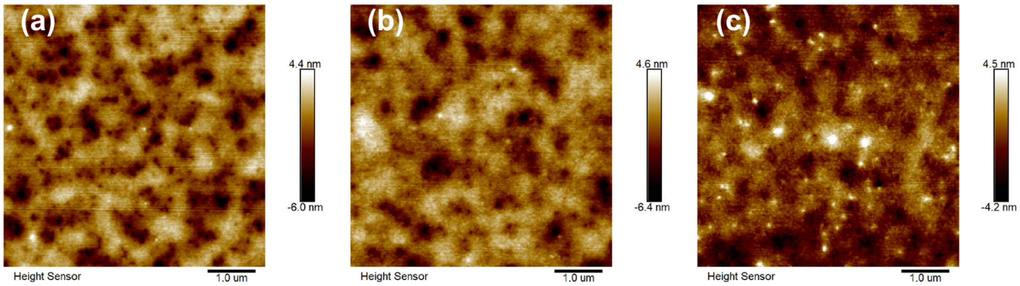

Figure S17 AFM height maps (5.5 μm×5.5 μm) of films (a) M1, (b) M3 and (c) M4.

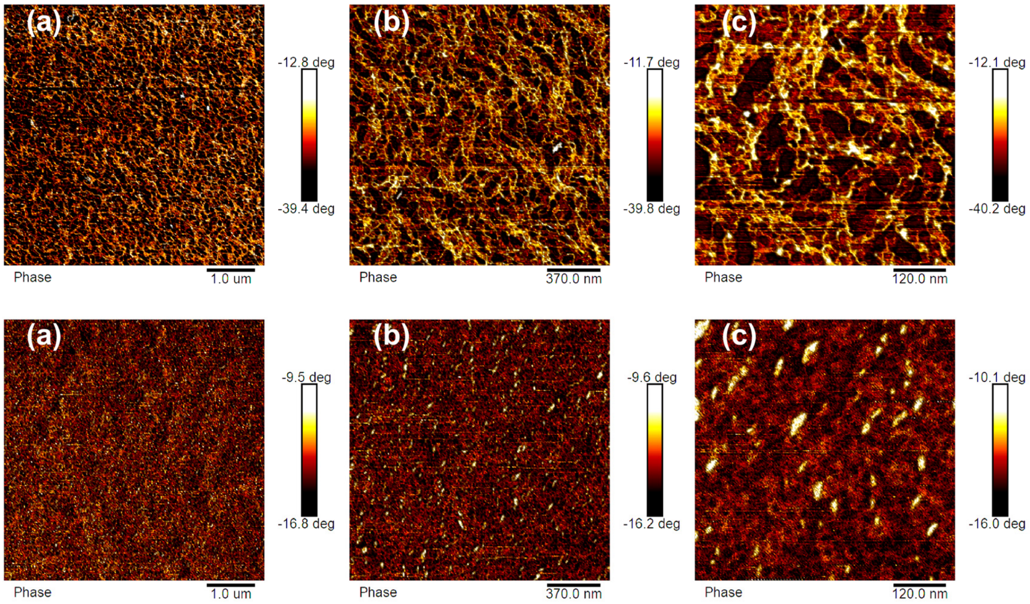

Figure S18 AFM Phase Image of films M3 (top) and M4 (bottom): (a) 5.51 μm×5.51 μm; (b) 1.83 μm×1.83 μm; (c) 608 nm×608 nm.

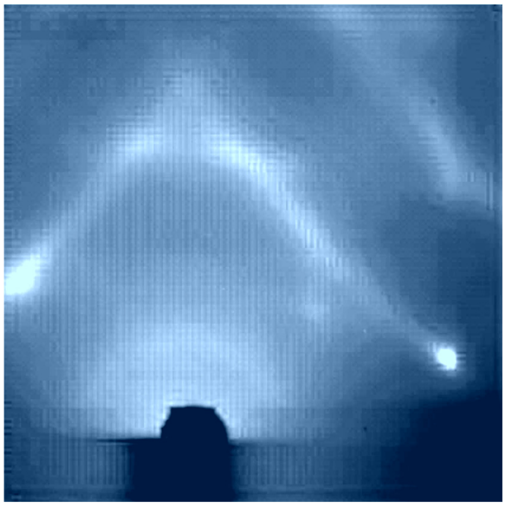

Figure S19 XRD analysis of thin film M4.

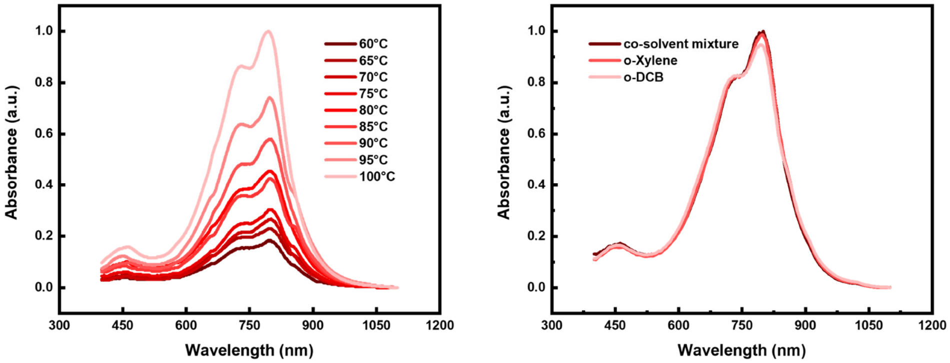

Figure 20 UV-Vis analysis of films prepared in the same solvent system (left), different solvent systems (right).

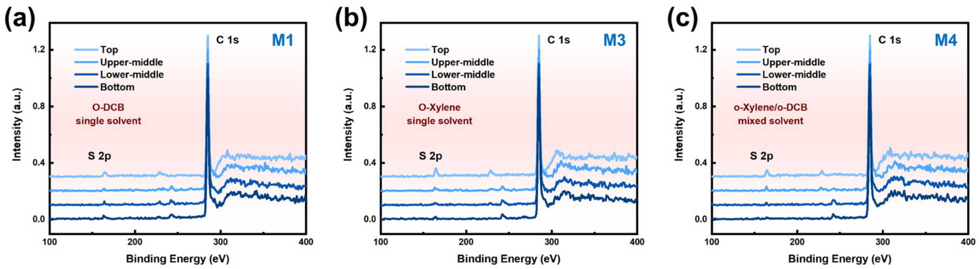

Figure S21 XPS analysis of films (a) M1, (b) M3 and (c) M4.

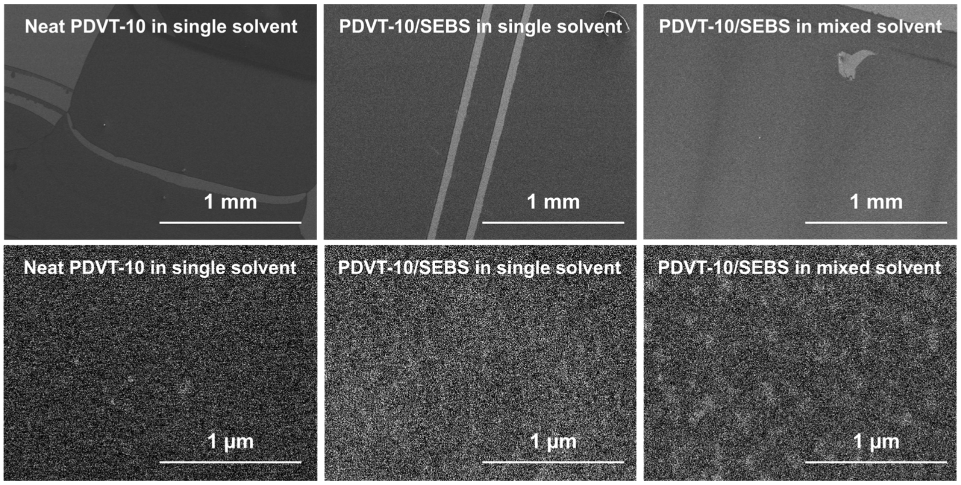

Figure S22 XPS analysis of films

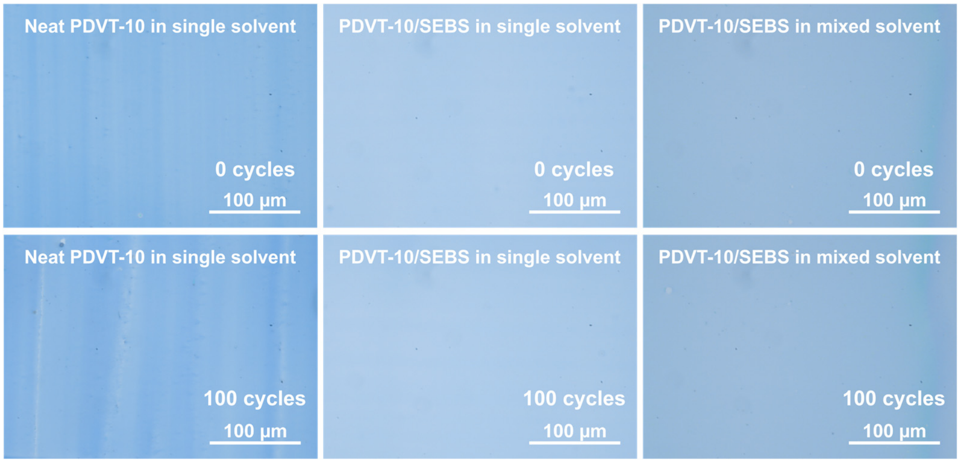

Figure S23 XPS analysis of films.

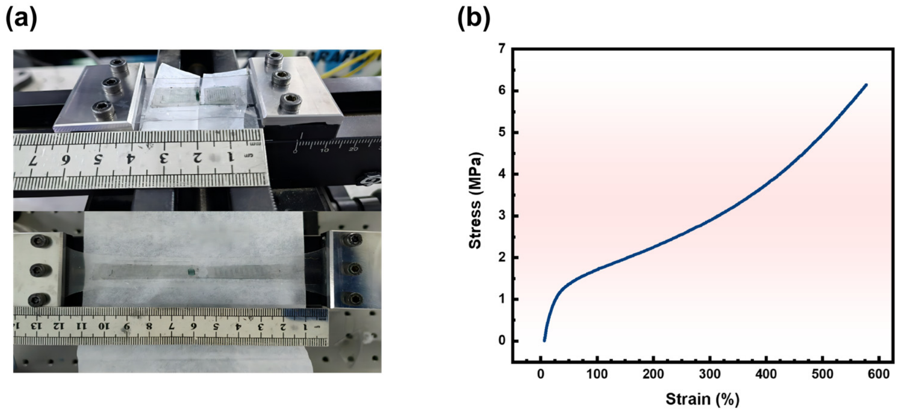

Figure S24 Mechanical tensile properties of fully stretchable OFETs: (left) images at 0% and 200% strain; (right) tensile stress-strain curve.

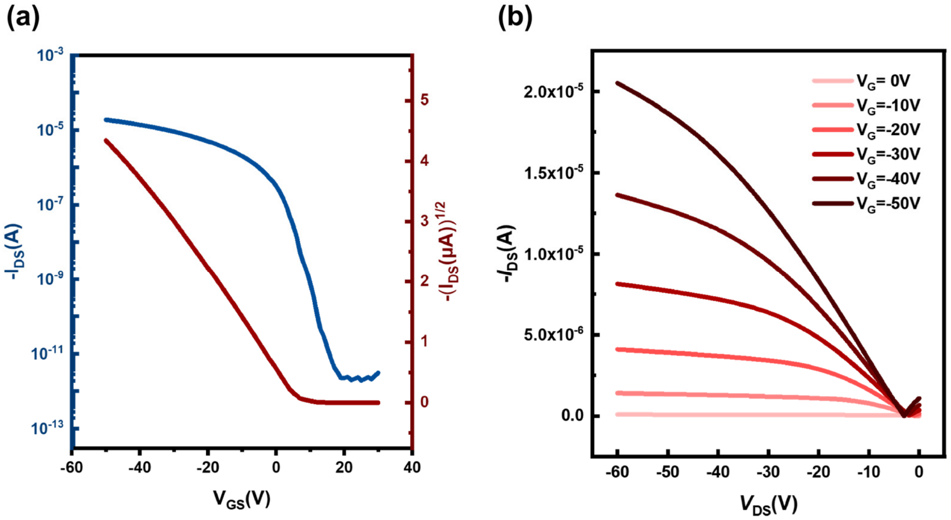

Figure S25 Basic electrical properties of a fully stretchable OFET: (left) transfer profile; (right) output profile.

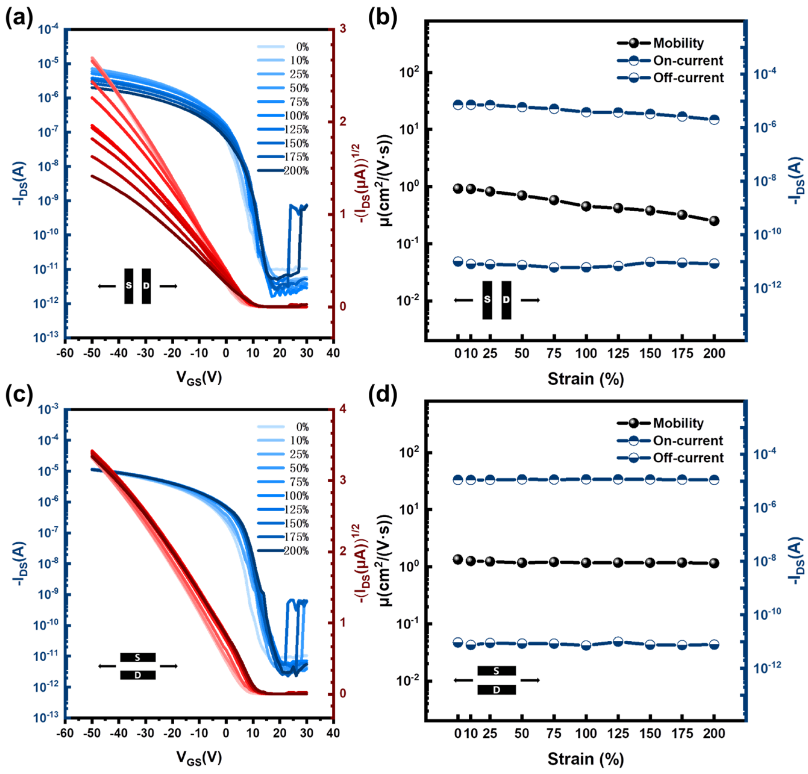

Figure S26 Mechanical-electrical properties of fully stretchable OFETs: (a) transfer curves obtained from the D3 device under different strains (TD // CTD); (b) changes in on/off current and mobility obtained from the D3 device under different strains (TD // CTD); (c) transfer curves obtained from the D3 device under different strains (TD⊥CTD); (f) changes in on/off current and mobility obtained from the D3 device under different strains (TD⊥CTD).

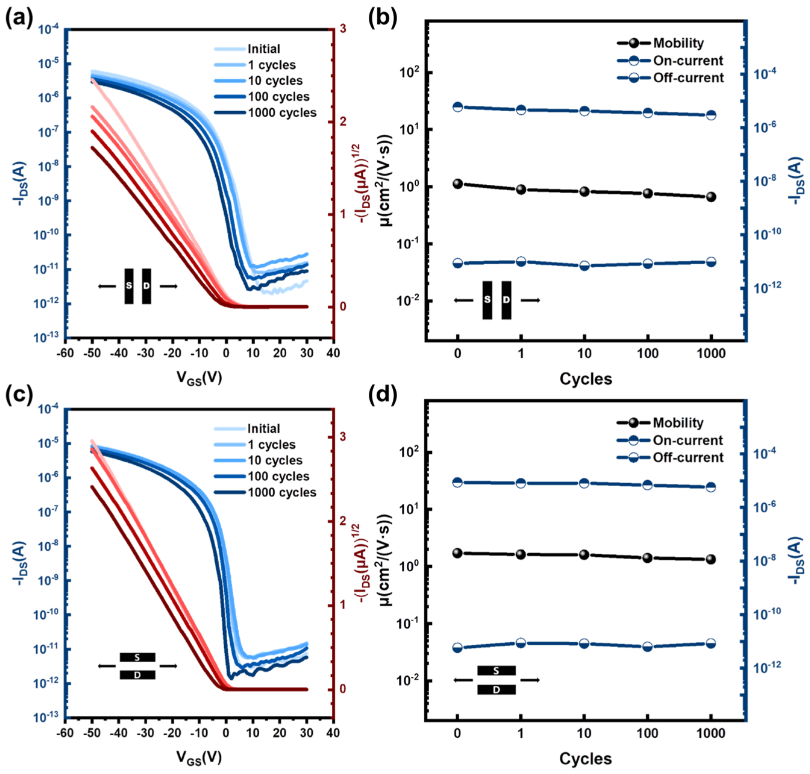

Figure S27 Mechanical-electrical properties of fully stretchable OFETs: (a) transfer curves obtained from the D3 device after multiple stretching-releasing cycles (up to 1000 cycles) at 100% strain (TD // CTD); (b) changes in on/off current and mobility obtained from the D3 device after multiple stretching-releasing cycles (up to 1000 cycles) at 100% strain (TD // CTD); (c) transfer curves obtained from the D3 device after multiple stretching-releasing cycles (up to 1000 cycles) at 100% strain (TD⊥CTD); (f) changes in on/off current and mobility obtained from the D3 device after multiple stretching-releasing cycles (up to 1000 cycles) at 100% strain (TD⊥CTD).

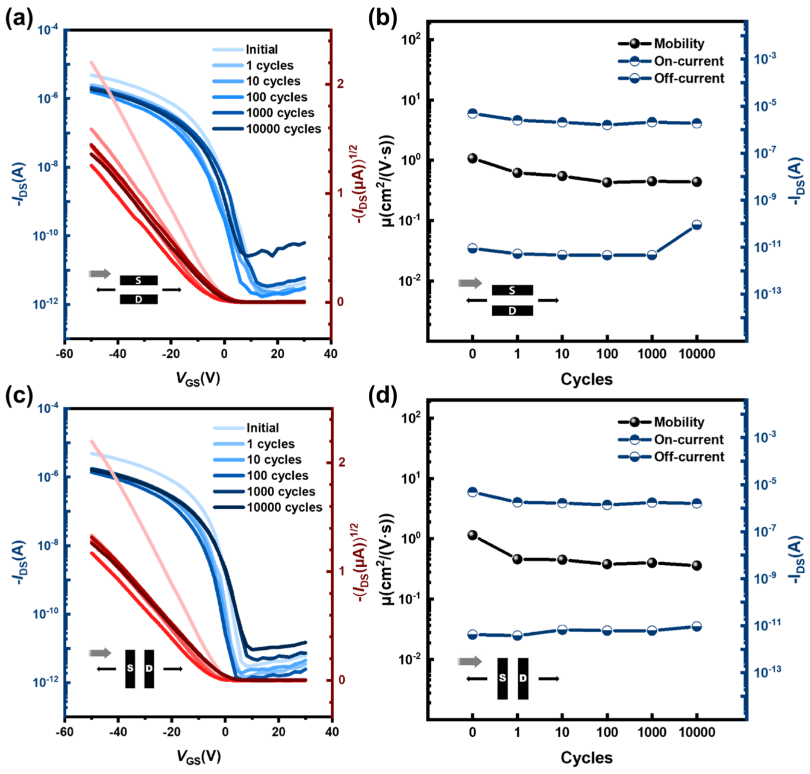

Figure S28 Mechanical-electrical properties of fully stretchable OFETs: (a) transfer curves obtained from the D4 device after multiple stretching-releasing cycles (up to 10000 cycles) at 100% strain (TD // CTD); (b) changes in on/off current and mobility obtained from the D4 device after multiple stretching-releasing cycles (up to 10000 cycles) at 100% strain (TD // CTD); (c) transfer curves obtained from the D4 device after multiple stretching-releasing cycles (up to 10000 cycles) at 100% strain (TD⊥CTD); (f) changes in on/off current and mobility obtained from the D4 device after multiple stretching-releasing cycles (up to 10000 cycles) at 100% strain (TD⊥CTD).

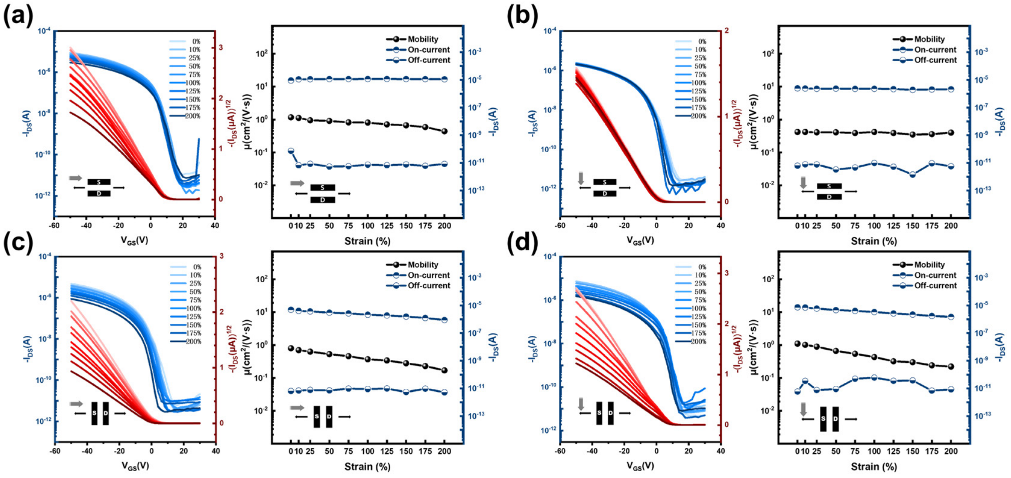

Figure S29 Transfer curves and changes in on/off current and mobility obtained from the D4 device under different strains: (a) TD∥SSD, TD⊥CTD; (b) TD⊥SSD, TD⊥CTD; (c) TD∥SSD, TD∥CTD; (d) TD⊥SSD, TD∥CTD. The inset shows the relationship between the stretching direction, charge transport direction, and solution shearing direction.

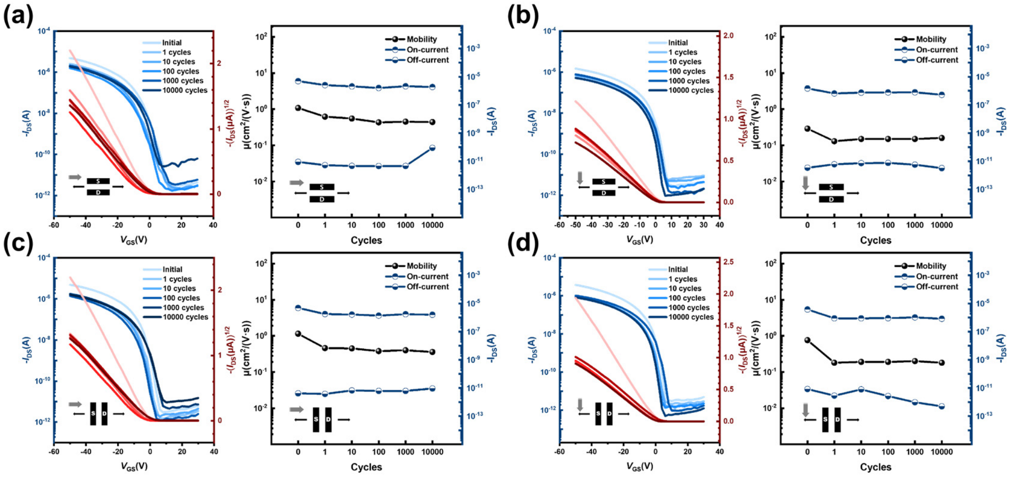

Figure S30 Transfer curves and changes in on/off current and mobility obtained from the D4 device after multiple stretching-releasing cycles (up to 10000 cycles) at 100% strain: (a) TD∥SSD, TD⊥CTD; (b) TD⊥SSD, TD⊥CTD; (c) TD∥SSD, TD∥CTD; (d) TD⊥SSD, TD∥CTD. The inset shows the relationship between the stretching direction, charge transport direction, and solution shearing direction.

Figure S31 illustrates the polymer PDVT-10 polymer molecular repeating units (**Figure S31a**), molecular structure (**Figure S31b**), conformation (**Figure S31c**), molecular recognition (non-covalent interactions between multiple molecular chains) (**Figure S31d**), stacking structure (Figure S31e), aggregation state of the polymer in the elastomers after film formation (**Figure S31f**), phase-separated structure (Figure S31g) with the macroscopic planar structure under OM (**Fig. S31h**). The efficient strain energy dissipation mechanism balances its charge transport over multiple length scales. The centre of **Figure S31** illustrates the mechanical energy dissipation mechanism of D-A polymer in OSC under strain conditions and a schematic of PDVT-10/SEBS OSC stretching on a flexible substrate. Including (i) molecular rotation and chain elongation. (ii) Sliding and alignment of polymer chains in the amorphous region, and reorientation and alignment of crystals embedded in the amorphous region with respect to the strain direction. (iii) Fracture of non-covalent cross-linking sites (e.g. H-bonds or metal coordination). (iv) Grain fracture, which ultimately leads to chemical bond breakage. During the stretching of PDVT-10/SEBS OSC, mechanical energy can be dissipated as a result without causing a significant decrease in charge transport properties.

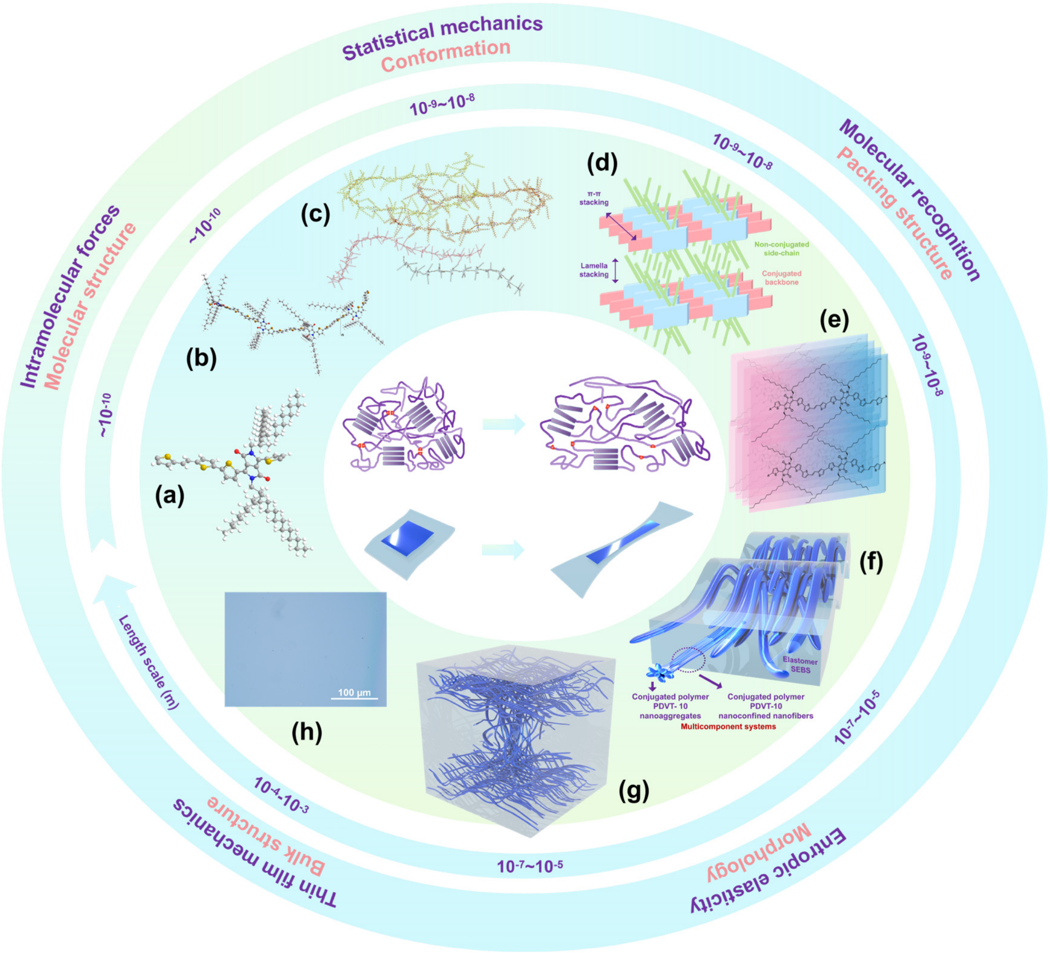

Figure S31 Design of stretchable PDVT-10/SEBS OSC and its chemical structural framework for controlling mechanical energy storage or dissipation: (centre) Mechanical energy dissipation mechanism of D-A polymer in OSC under strain conditions and schematic diagram of stretchable PDVT-10/SEBS OSC on flexible substrate. (a) Chemical structure of the molecular repeating unit of PDVT-10 polymer. (b) Molecular composition of PDVT-10 polymer molecule. (c) Conformation of PDVT-10 polymer molecule in SEBS. (d) Molecular recognition between multiple PDVT-10 polymer molecules. (e) Crystal stacking structure of PDVT-10 polymer in elastomer matrix SEBS. (f) Nano-limited domain effect in PDVT-10/SEBS OSC. (g) Phase separation structure in PDVT-10/SEBS OSC. (h) OM image of PDVT-10/SEBS OSC.

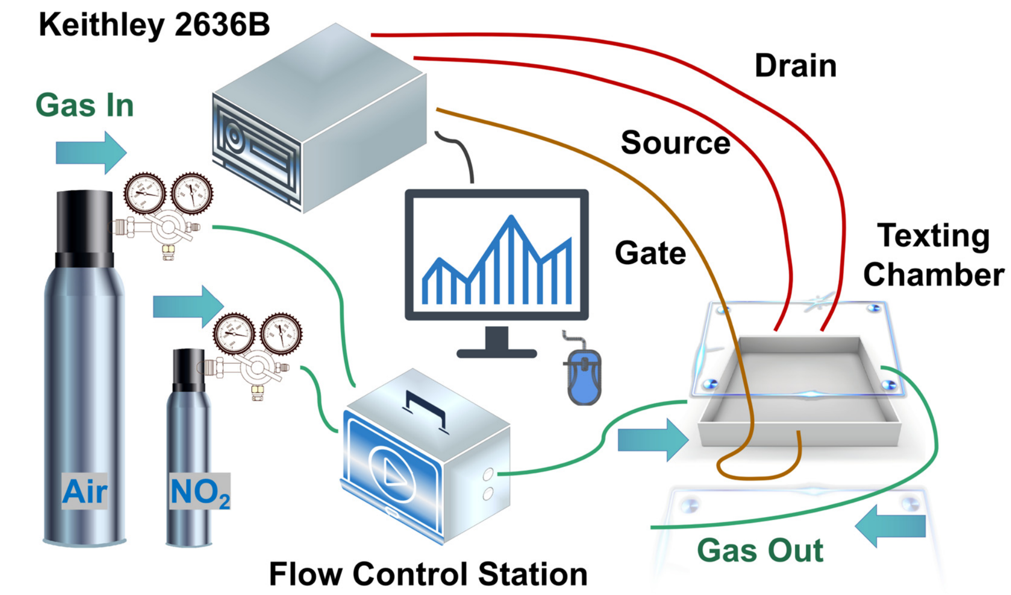

Figure S32 Schematic diagram of the toxic small-molecule gas sensing evaluation system.

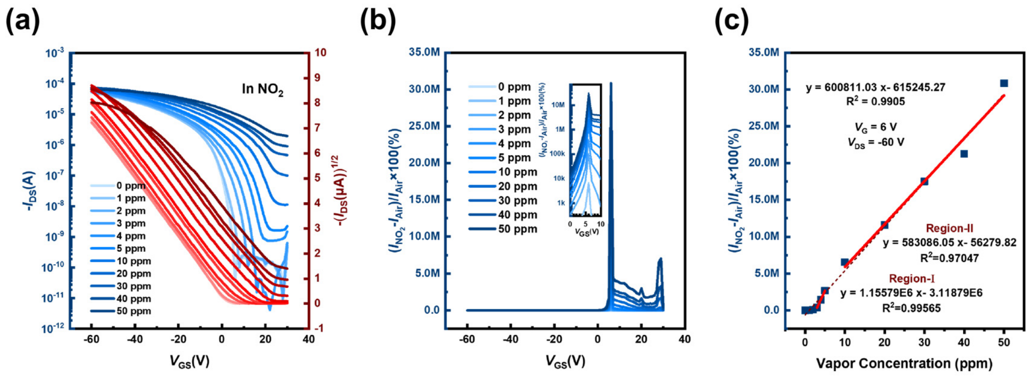

Figure S33 Sensing performance of the D4' sensor toward various concentrations of NO_2: (a) Transfer characteristics; (b) Variation of gas responsivity with gate voltage; (c) Linear plot of responsivity vs NO_2 concentration. The sensitivity data are calculated from the transfer characteristics (current from the subthreshold region).

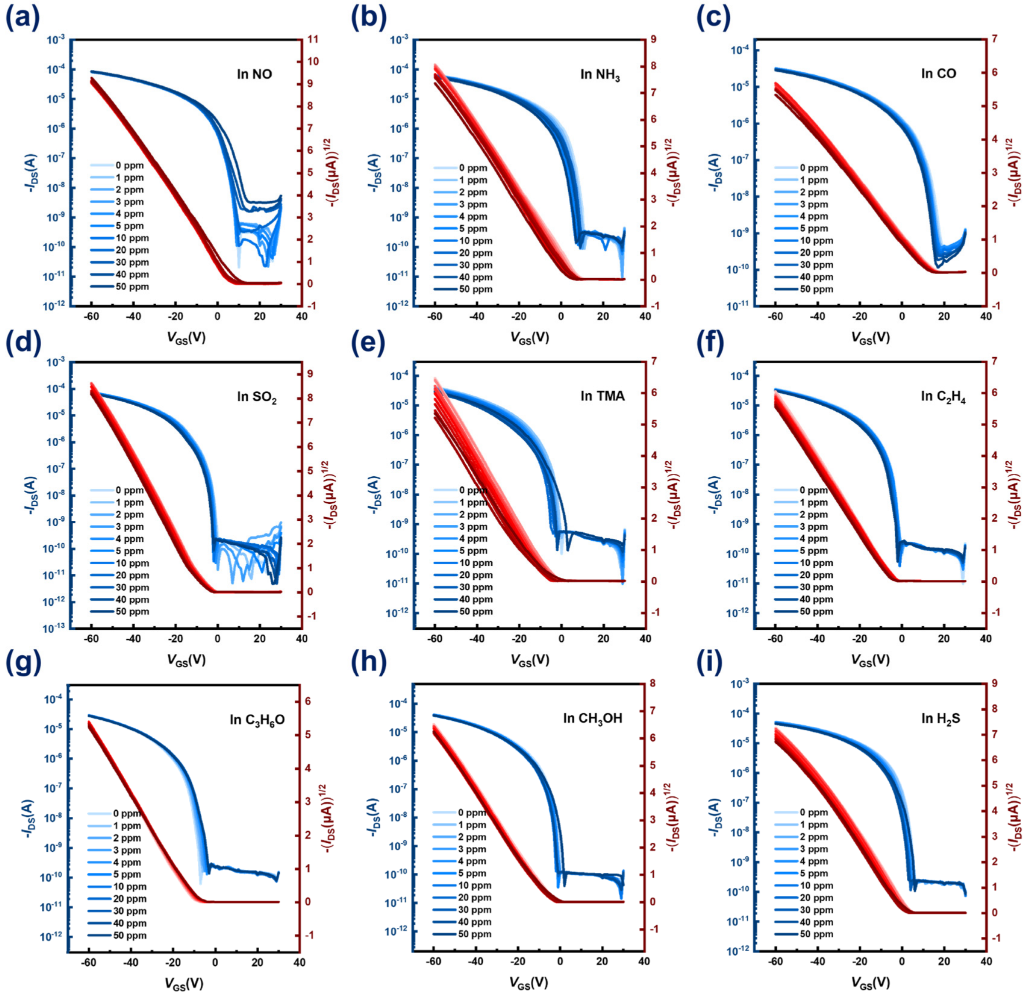

Figure S34 Transfer characteristics of the D4' sensor toward various concentrations of (a) NO, (b) NH_3, (c) CO, (d) SO_2, (e) TMA, (f) C_2H_4, (g) C_2H_6O, (h) CH_3OH and (i) H_2S.

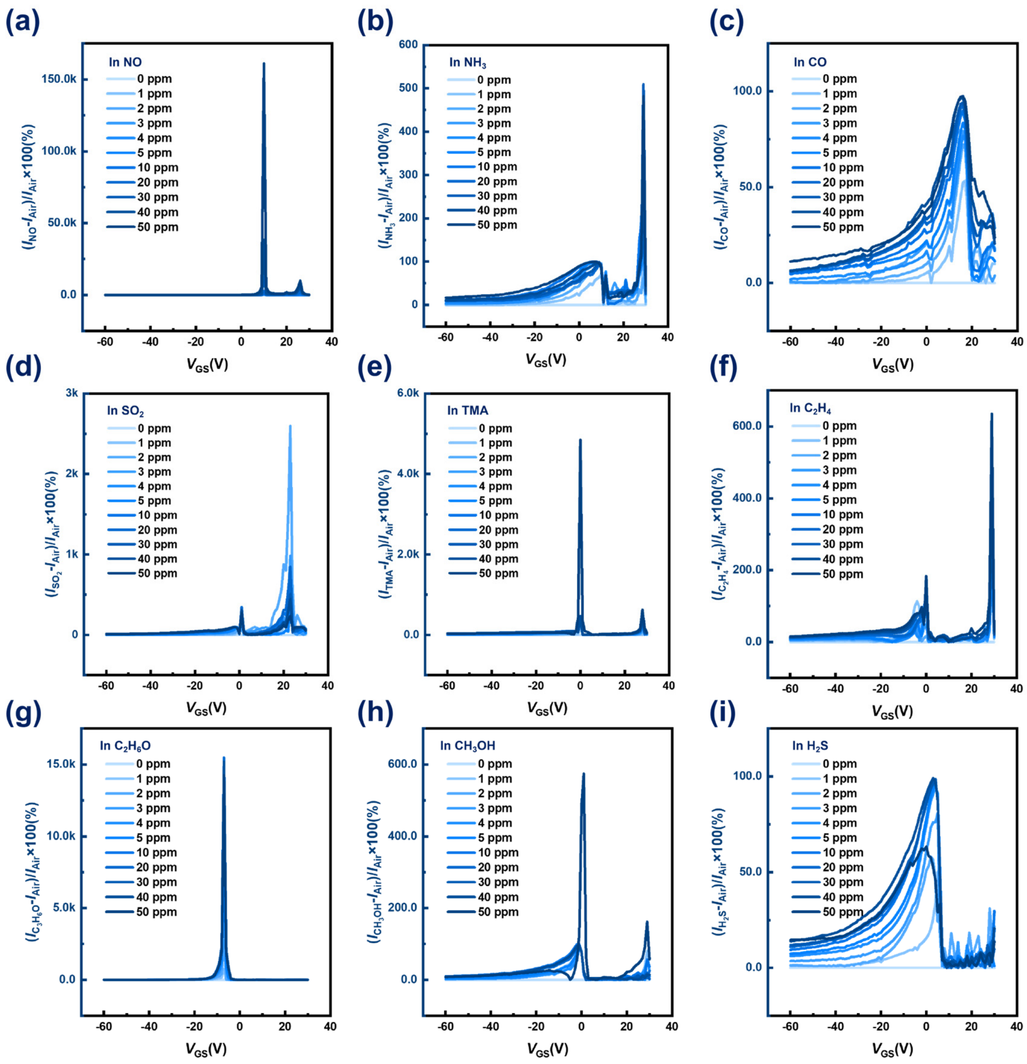

Figure S35 Variation of gas responsivity with gate voltage of the D4' sensor toward various concentrations of (a) NO, (b) NH_3, (c) CO, (d) SO_2, (e) TMA, (f) C_2H_4, (g) C_2H_6O, (h) CH_3OH and (i) H_2S.

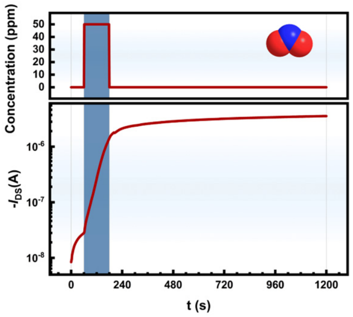

Figure S36 Transient analysis of the D4' sensor toward various concentrations of NO_2.

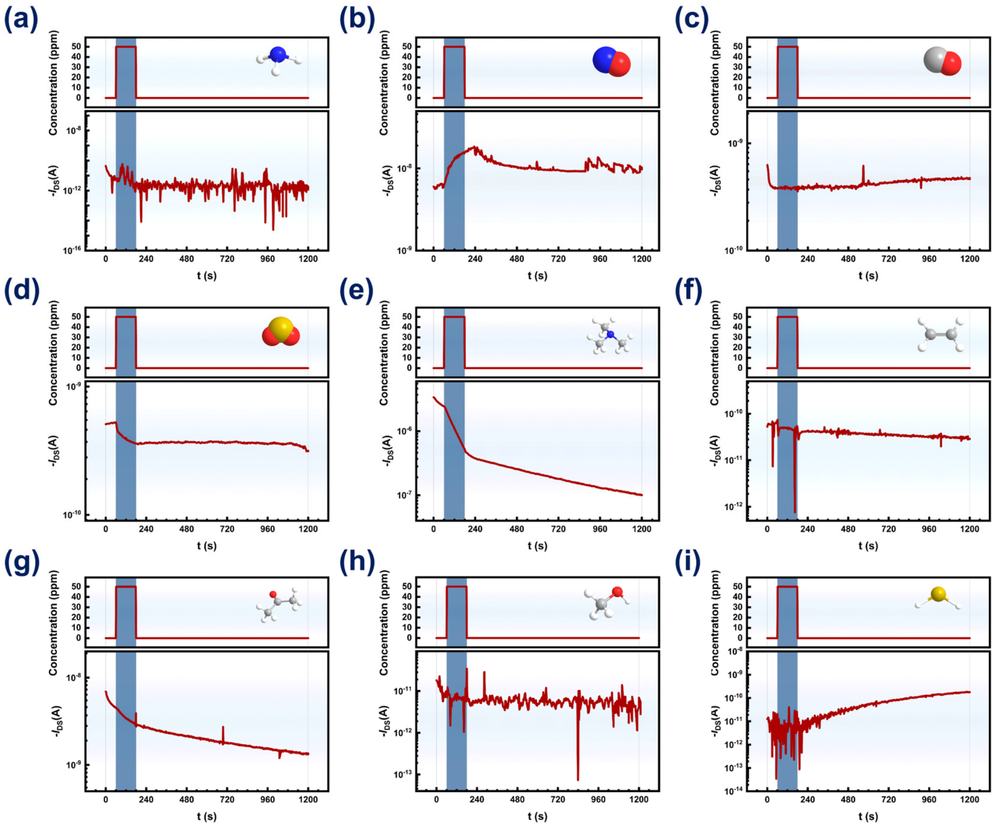

Figure S37 Transient analysis of the D4' sensor toward various concentrations of (a) NO, (b) NH_3, (c) CO, (d) SO_2, (e) TMA, (f) C_2H_4, (g) C_2H_6O, (h) CH_3OH and (i) H_2S.

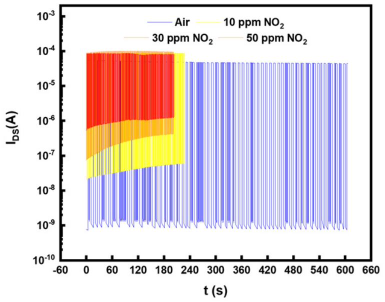

Figure S38 Transient analysis under constant voltage step output of the D4' sensor toward various concentrations of NO_2.

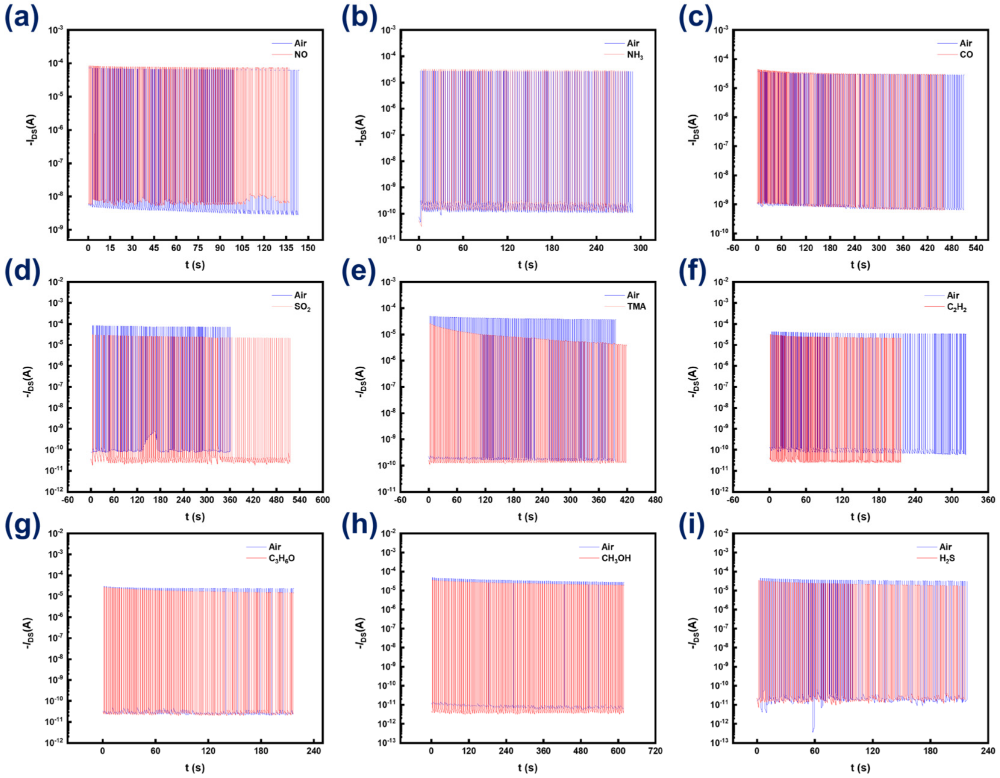

Figure S39 Transient analysis under constant voltage step output of the D4' sensor toward various concentrations of (a) NO, (b) NH_3, (c) CO, (d) SO_2, (e) TMA, (f) C_2H_4, (g) C_2H_6O, (h) CH_3OH and (i) H_2S.

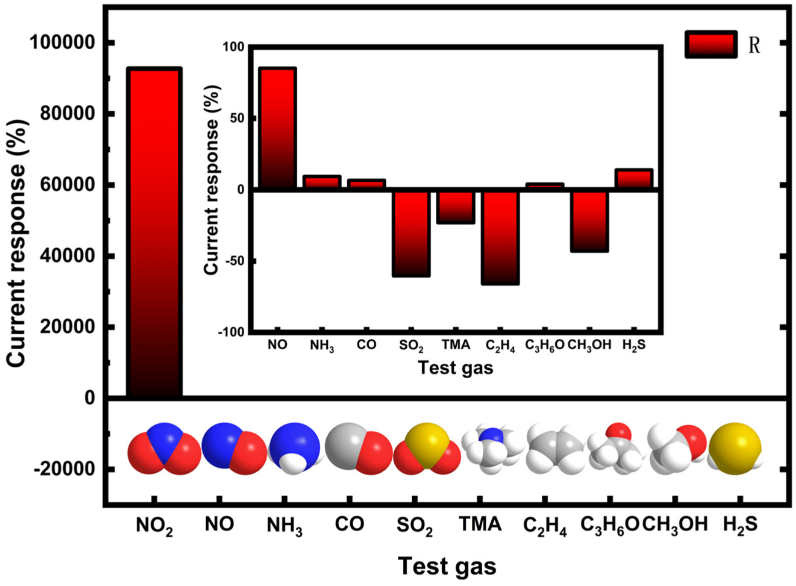

Figure S40 Selectivity behavior of the D4' sensor tested against the same concentration (50 ppm) of various toxic small molecule gas analytes.

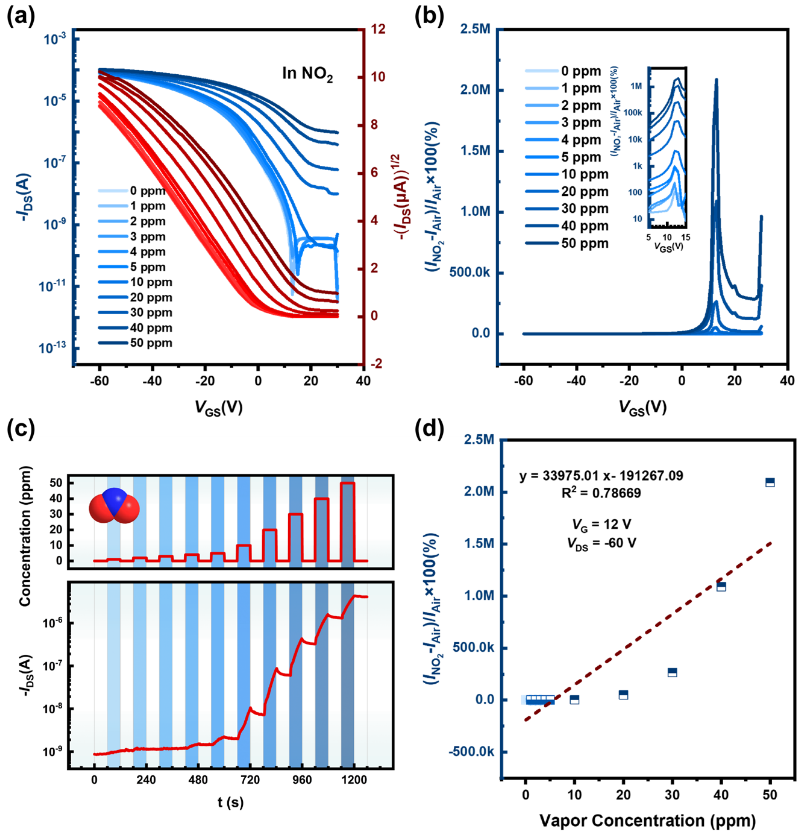

Figure S41 Sensing performance of the D3' sensor toward various concentrations of NO_2: (a) Transfer characteristics; (b) Variation of gas responsivity with gate voltage; (c) Transient analysis; (d) Linear plot of responsivity vs NO_2 concentration.The sensitivity data are calculated from the transfer characteristics (current from the subthreshold region).

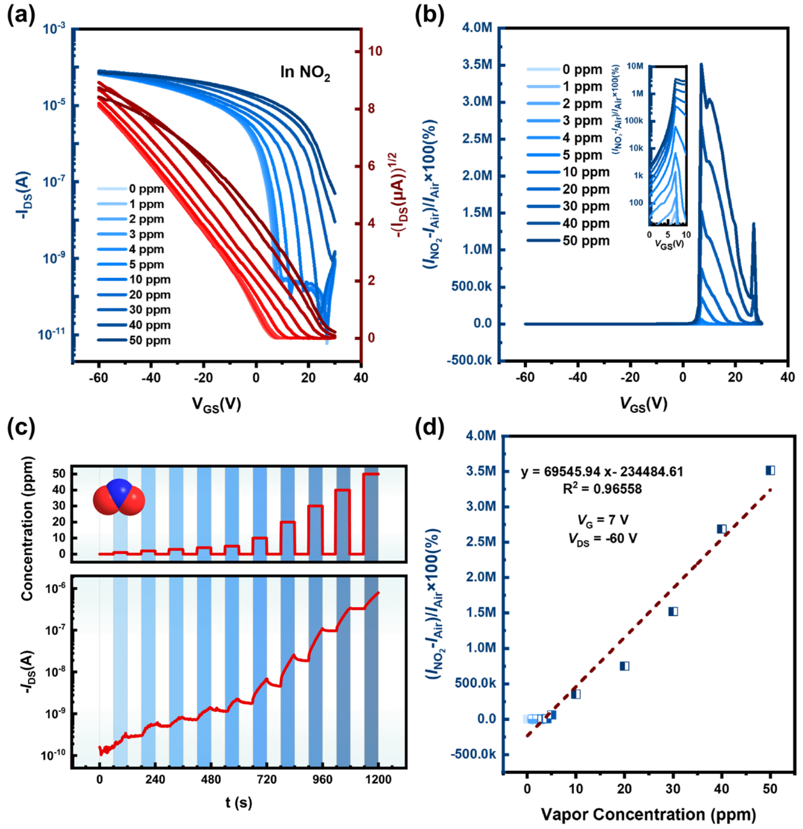

Figure S42 Sensing performance of the D4' sensor toward various concentrations of NO_2: (a) Transfer characteristics; (b) Variation of gas responsivity with gate voltage; (c) Transient analysis; (d) Linear plot of responsivity vs NO_2 concentration.The sensitivity data are calculated from the transfer characteristics (current from the subthreshold region).

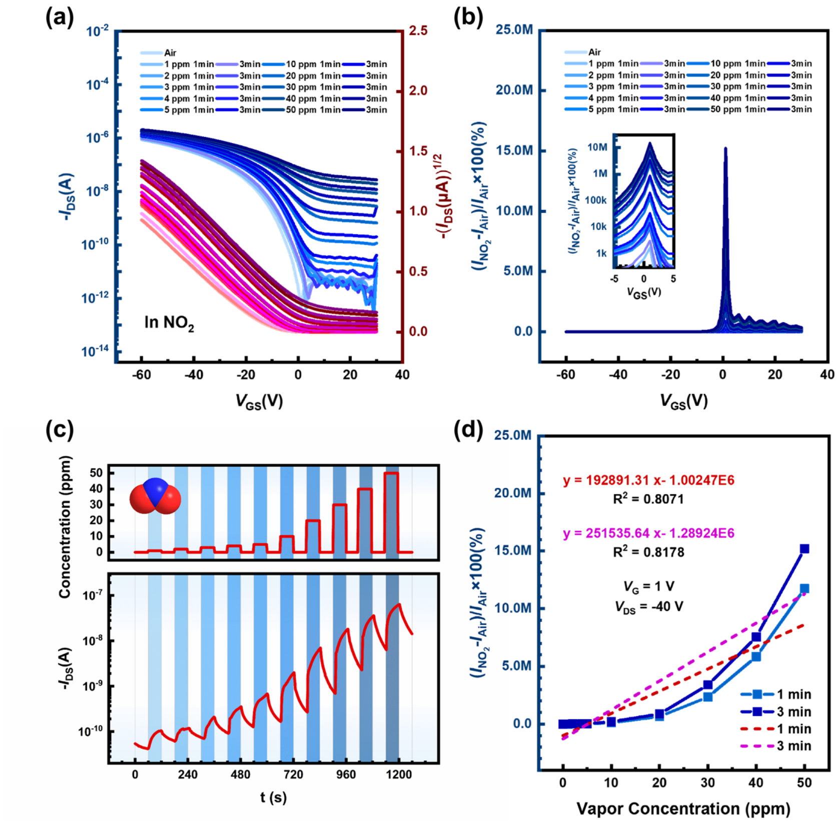

Figure S43 Sensing performance of the D3 sensor toward various concentrations of NO_2: (a) Transfer characteristics; (b) Variation of gas responsivity with gate voltage; (c) Transient analysis; (d) Linear plot of responsivity vs NO_2 concentration.The sensitivity data are calculated from the transfer characteristics (current from the subthreshold region).

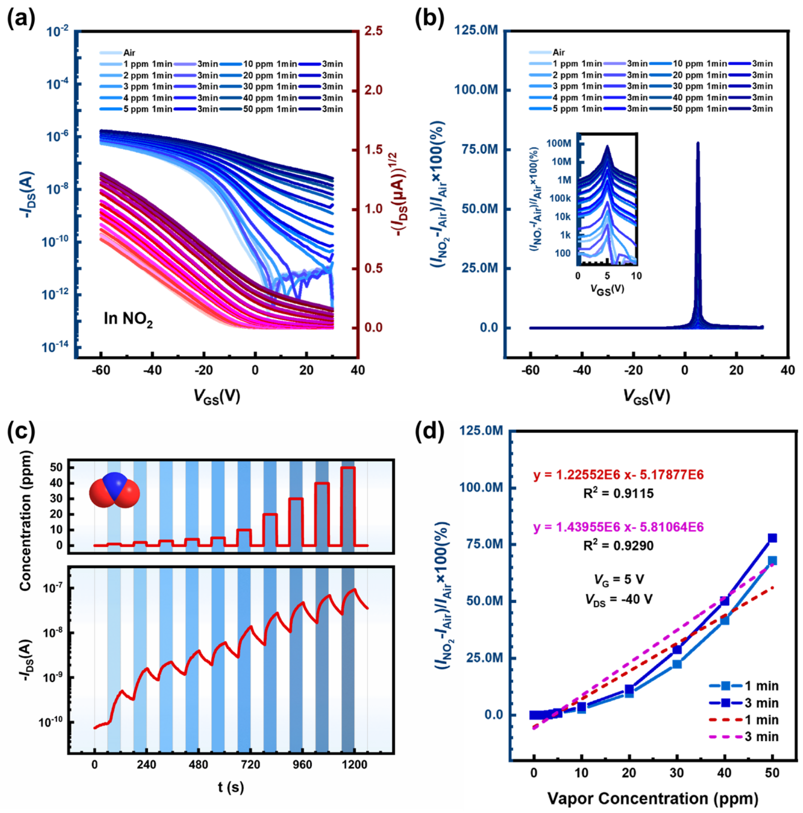

Figure S44 Sensing performance of the D4 sensor toward various concentrations of NO_2 : (a) Transfer characteristics; (b) Variation of gas responsivity with gate voltage; (c) Transient analysis; (d) Linear plot of responsivity vs NO_2 concentration.The sensitivity data are calculated from the transfer characteristics (current from the subthreshold region).

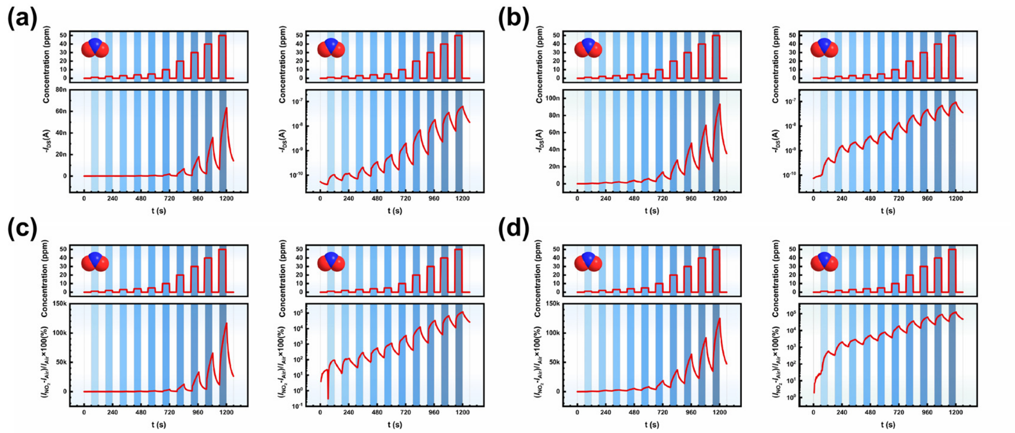

Figure S45 Sensing performance of fully stretchable OFETs toward various concentrations of NO_2 : (a) Transient analysis of the D3 sensor (Linear vs. Logarithmic Axes). (b) Transient analysis of the D4 sensor (Linear vs. Logarithmic Axes); (c) Real-time responsivity obtained from the D3 sensor (Linear vs. Logarithmic Axes); (d) Real-time responsivity obtained from the D4 sensor (Linear vs. Logarithmic Axes).

Table S4. Comparison previously reported gas sensing characteristics of various NO_2 sensors.

**Parameters**	**OSC Materials**	**Mobility ($cm^2\ V^{-1}\ s^{-1}$)**	**Responsivity**	**Mechanical-Electrical Performance**
This work	M3	2.51	15.2×10^6 % (50 ppm, 3 min)	The mobility is optimally maintained at 90% under 100% strain
This work	M4	2.71	77.9×10^6 % (50 ppm, 3 min)	The mobility is optimally maintained at 70% under 100% strain
[1]	CuPc	4.9×10^{-4}	1.6×10^5 % (30 ppm, 2 min)	/
[2]	CuPc/Pentacene	0.13	30% (15 ppm, 5 min)	/
[3]	Mechanically rubbed Pentacene	0.09	1.2% (10 ppm, 2 min)	/
[4]	PDQT/FPPTS	0.13	780% (30 ppm, 2 min)	/
[5]	PS/PMMA	/	7633% (30 ppm, 10 min)	/

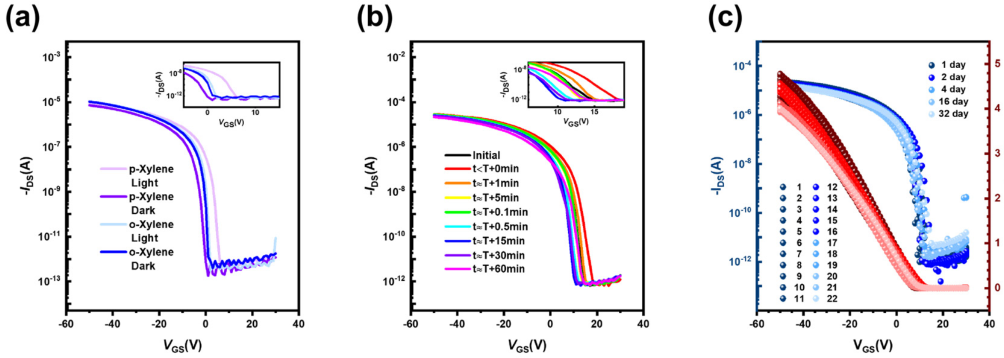

Figure S46 Environmental and Operational Stability of the OFET device D4' with rigid substrate.

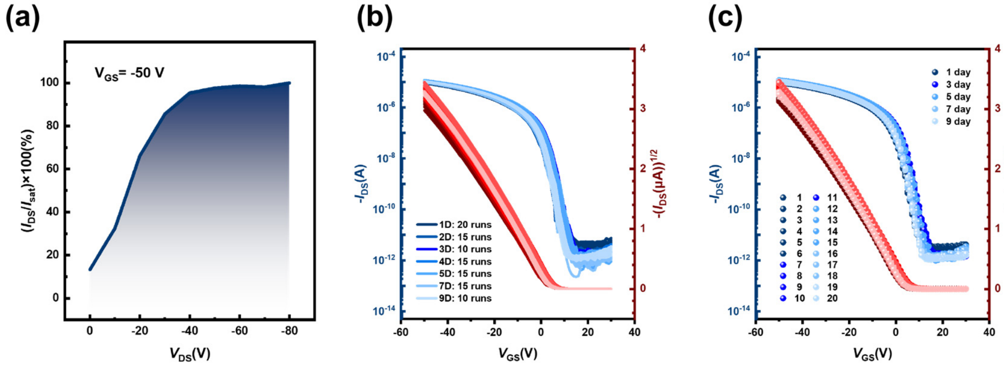

Figure S47 Environmental and Operational Stability of the Fully Stretchable OFET Device D4 with flexible substrate.

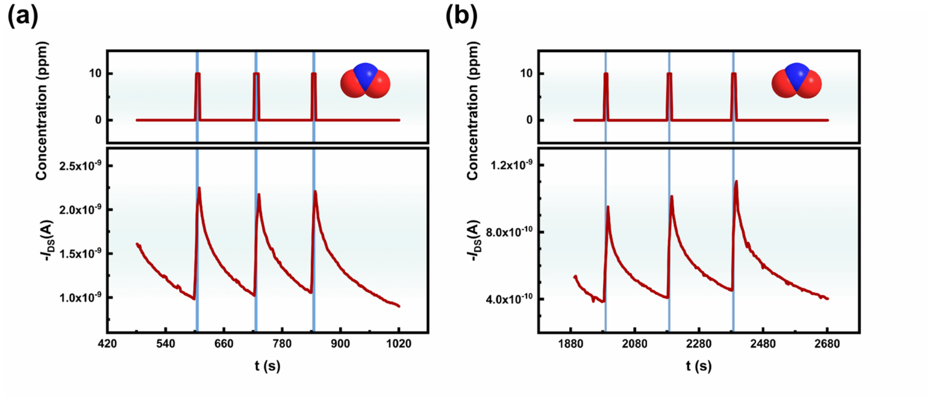

Figure S48 Repeatability study of (a) the D3 sensor and (b) the D4 sensor tested at 10 ppm NO_2 concentration repeated for 3 cycles.

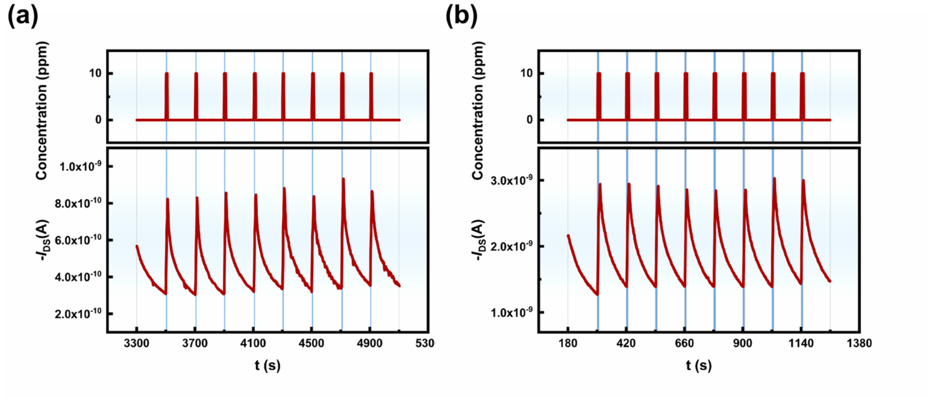

Figure S49 Repeatability study of (a) the D3 sensor and (b) the D4 sensor tested at 10 ppm NO_2 concentration repeated for 8 cycles after 5h of continuous testing.

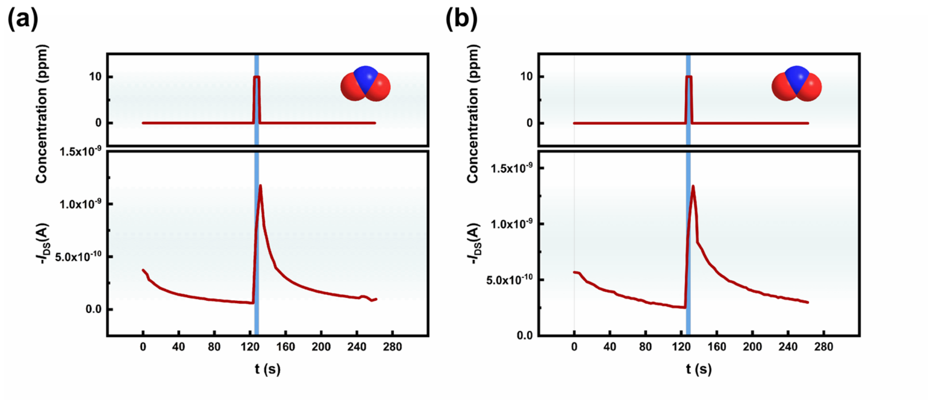

Figure S50 Response and recovery curve obtained from (a) the D3 sensor and (b) the D4 sensor in the presence of 10 ppm NO_2 gas.

References

1. Huang, W.; Zhuang, X.; Melkonyan, F.S.; Wang, B.; Zeng, L.; Wang, G.; Han, S.; Bedzyk, M.J.; Yu, J.; Marks, T.J.; et al. UV–Ozone Interfacial Modification in Organic Transistors for High-Sensitivity NO_2 Detection. *Adv. Mater.* **2017**, *29*, 1701706.
2. Han, S.; Cheng, J.; Fan, H.; Yu, J.; Li, L. Achievement of High-Response Organic Field-Effect Transistor NO_2 Sensor by Using the Synergistic Effect of ZnO/PMMA Hybrid Dielectric and CuPc/Pentacene Heterojunction. *Sensors* **2016**, *16*, 1763.
3. Zeng, Y.; Huang, W.; Shi, W.; Yu, J. Enhanced sensing performance of nitrogen dioxide sensor based on organic field-effect transistor with mechanically rubbed pentacene active layer. *Appl. Phys. A* **2015**, *118*, 1279–1285.
4. Song, R.; Zhou, X.; Wang, Z.; Zhu, L.; Lu, J.; Xue, D.; Wang, Z.; Huang, L.; Chi, L. High selective gas sensors based on surface modified polymer transistor. *Org. Electron.* **2021**, *91*, 106083.
5. Zhu, Y.; Dong, J.; Li, G.; Liu, C.; Xie, Q.; Wang, L.; Wang, L.J.; You, M. Bilayer polymer dielectric systems for high response NO_2 gas sensors. *Mater. Lett.* **2021**, *288*, 129370.
